# Empirical challenges from the comparative and developmental literature to the Shared Intentionality Theory – a review of alternative data on recursive mind reading, prosociality, imitation and cumulative culture

**DOI:** 10.3389/fpsyg.2023.1157137

**Published:** 2023-10-13

**Authors:** Gabriela-Alina Sauciuc, Tomas Persson

**Affiliations:** Department of Philosophy/Cognitive Science, Lund University, Lund, Sweden

**Keywords:** cooperation, joint attention, false belief, resource sharing, altruism and prosocial behaviour, socio-ecological factors, great apes, children

## Abstract

Humans have an irresistible inclination to coordinate actions with others, leading to species-unique forms of cooperation. According to the highly influential Shared Intentionality Theory (SITh), human cooperation is made possible by shared intentionality (SI), typically defined as a suite of socio-cognitive and motivational traits for sharing psychological states with others, thereby enabling individuals to engage in joint action in the mutually aware pursuit of shared goals. SITh theorises that SI evolved as late as 400,000 years ago, when our ancestors (in particular, *Homo heidelbergensis*) turned to a kind of food procurement that obligatorily required joint coordinated action. SI is, thus, hypothesized to be absent in other extant species, including our closest genetic relatives, the nonhuman great apes (“apes”). According to SITh, ape psychology is exclusively driven by individualistic motivations, as opposed to human psychology which is uniquely driven by altruistic motivations. The evolutionary scenario proposed by SITh builds on a series of findings from socio-cognitive research with apes and human children, and on the assumption that abilities expressed early in human development are human universals, unlikely to have been shaped by socio-cultural influences. Drawing on the primatological and developmental literature, we provide a systematic – albeit selective – review of SITh-inconsistent findings concerning psychological and behavioural traits theorised to be constitutive of SI. The findings we review pertain to all three thematic clusters typically addressed in SITh: (i) recursive mind reading; (ii) prosociality; (iii) imitation and cumulative culture. We conclude that such alternative data undermine two core SITh claims: the late evolutionary emergence of SI and the radical divide between ape and human psychology. We also discuss several conceptual and methodological limitations that currently hamper reliable comparative research on SI, in particular those engendered by Western-centric biases in the social sciences, where an overreliance on Western samples has promoted the formulation of Western-centric conceptualisations, operationalisations and methodologies.

## 1. Introduction

From passing a ball to each other to outstanding joint achievements such as the International Space Station, cooperation is central in order to succeed. While it might seem ordinary to us, human cooperation is remarkable from an evolutionary perspective, given its scope and flexibility, and especially our natural - and irresistible - inclination to coordinate actions with others. Whether directed at accomplishing a task (e.g., moving a table with someone), or exerted for leisurely purposes (e.g., dancing tango), joint coordinated action is uniquely ubiquitous in our species. But where does this uniqueness start?

According to a currently dominant evolutionary theory, cooperation and joint action are made possible by a purportedly human-specific kind of psychology, based on *shared intentionality* – in short SI (e.g., Tomasello et al., 2012; Tomasello, 2014, 2018a, 2022). In this view, SI captures ‘skills and motivations’ that promote shared psychological states with others, which in turn enables individuals to engage in collaborative interactions, in the (mutually aware) pursuit of shared goals. If I do not grasp and share your playful motivation when you throw a ball to me, we might end up in conflict instead of cooperating to play. According to this theory, SI is hypothesised to have emerged as late as 400,000 years ago, when Middle Pleistocene hominins (specifically *Homo heidelbergensis*) would have turned to a kind of food procurement based on active and obligate cooperation, which required joint coordinated action (e.g., Tomasello et al., 2012; Tomasello, 2014, 2018a,b). SI is, thus, considered to be absent in other species, including the non-human great apes (henceforth apes), whose psychology is claimed to be driven by individualistic motivations as opposed to altruistic motivations which are seen as specific to humans. The radical divide between human and ape psychology postulated by this *Shared Intentionality Theory* (henceforth SITh), entails a lack of phylogenetic continuity and an absence of relevant precursor traits, thereby suggesting that research aimed at investigating the presence of such traits in non-human species may be meaningless.

In the present – and selective – review we target the empirical foundations of SITh by discussing alternative data from great ape studies, which are inconsistent with SITh claims regarding most of the currently proposed criteria for SI. Since SITh evolutionary arguments build on performance contrasts between apes and human children, our review will also cover some developmental data that are inconsistent with SITh. We first provide a summary of the putative evolutionary scenario outlined in SITh (Section 2) and then proceed to reviewing SITh-inconsistent findings in Sections 3–5. Since the case for SITh builds on data that spans three broad thematic clusters, each review section will focus on a specific cluster. In Section 3, we will review SITh-inconsistent data pertaining to the ‘recursive mind reading’ cluster, in particular data concerning joint attention (Section 3.1) and the understanding of others’ beliefs (Section 3.2) in apes and human development. In Section 4, we review SITh-inconsistent data pertaining to the ‘prosociality’ cluster, in particular data on altruistic and cooperative behaviour, including instrumental helping (Section 4.1), resource sharing and cooperation (Section 4.2), as well as information sharing and the coordination of cooperative activities (Section 4.3). Section 4.2 is further divided into sub-sections covering findings from experimental (Section 4.2.1) and observational (Section 4.2.2) research on resource sharing in apes, data on mechanisms hypothesised to sustain and stabilise cooperation (Section 4.2.3), data indicating within-species variability and potential methodological concerns (Section 4.2.4.), as well as SITh-inconsistent developmental data (Section 4.2.5). Finally, in Section 5 we review and discuss findings related to the ‘imitation and cumulative culture’ cluster. In Section 6, then, we conclude with a plea for shifting comparative and developmental research in this area from a top-down strive to find behavioural matches to theoretical constructs whose operationalisation is currently vague, fluctuating and Western-centric, toward a bottom-up approach that examines joint behaviours against the background of contextual, individual, developmental, and socio-ecological variables. We also point out three significant biases that currently hamper reliable comparative work, and surmise that the currently available corpus of empirical evidence is instead consistent with a gradual view on the evolutionary emergence of behavioural and cognitive abilities that enable joint action and cooperation, rather than the seeming divide proposed by SITh.

We would like to clarify from the start that we do not claim that apes are capable of the same levels of joint understanding and cooperative feats as humans. Our aim is to give due exposure to the wealth of SITh-inconsistent empirical evidence since some of this evidence is absent in some of the SITh theoretical outlines cited above or, when present, tends to be treated (and dismissed) too summarily and selectively. This is important for at least two reasons. First, it is important to bring some nuance to views on the evolution of human cooperation in disciplines that tend to uncritically embrace SITh claims, even when these are only moderately supported by evidence. Given the influential status of SITh in many disciplines, SITh-consistent data currently enjoys disproportionately more visibility than SITh-inconsistent data. We hope that this review will help counterbalancing this trend. Second, it is important to counter the risks posed by the radical claims of SITh to continued research on SI-related abilities in apes. In the face of widespread claims that certain abilities are uniquely human, funding and carrying out research with nonhuman species on SI-related traits will appear unreasonable. To counter such risks, we also call for increased conceptual and terminological clarity, de-biased approaches in the field, and for duly considering precursors and continuity when it is warranted.

## 2. SITh: the putative evolutionary scenario of the emergence of ‘we’ intentionality

According to SITh (e.g., Tomasello et al., 2012; Tomasello, 2014, 2018a) climate changes forced hominins of Middle Pleistocene (i.e., *Homo heidelbergensis*) to turn to large game scavenging and hunting, which led to obligate interdependent foraging in small dyadic or triadic groups. Joint goal formation, it is argued, was necessary for such group activities, and thus obligatory for survival. It is further theorised that this putative scenario of obligate interdependence created selective pressures for action- and attention coordination, thereby leading to the emergence of evolutionarily novel forms of communication, such as pointing, iconic gestures, and pantomime. With such new skills postulated as vital, a novel social selection process is hypothesised to have emerged, that specifically selected for communicative skills and cooperative motives, and generated a novel concern with being (seen as) a good communicator and collaboration partner. In turn, this entailed increased self-monitoring of one’s own actions and a motivation to relate one’s actions to the perspective (and potential evaluations) of cooperation partners (Tomasello et al., 2012; Tomasello, 2014, 2018a).

The socio-cognitive outcome of these putative developments is hypothesised to be a radically new psychology dubbed *joint intentionality based on joint agency* (Tomasello, 2018a, p. 664), where collaboration partners had to anticipate each other’s perspective “which required socially recursive inferences that embedded the intentional stance of one partner within those of the other” (Tomasello, 2014, p. 72). A hypothetical characteristic of this psychology was also “a new kind of cooperative rationality” and a “kind of we > me morality” grounded in a sense of obligation to *joint commitments* and treating others with a sense of *fairness*, which in turn was derived from a sense of *self-other equivalence* (Tomasello, 2018a, pp. 664–665). *Joint commitment*, it is argued, was enabled by the newfound skills of cooperative communication and further entailed a mutual agreement that defection was deserving of punishment (Tomasello, 2018a, 2022). According to SITh, this type of reasoning gave rise to the emergence of moral emotions (guilt, shame) as an affective tool for regulating the maintenance of commitment and fair play (Tomasello, 2018a, 2022).

The second evolutionary leap toward human-specific cooperation according to SITh is the emergence of *collective intentionality* in *H. sapiens* (about 150 kya). This is theorised to be the result of selective pressures created by increased group size and intergroup competition. Such demographic conditions raised hypothetical novel challenges, such as having to collaborate with strangers that nevertheless belonged to one’s much enlarged group, thereby creating a need for ingroup identification. According to SITh, such challenges were initially solved by the conventionalisation of behavioural practices (e.g., ways to forage or to process food), which thus could serve as group identity markers (Tomasello et al., 2012; Tomasello, 2014, 2018a). As doing things in the conventional way allegedly became mandatory for survival, conformity and teaching ensued as socio-cognitive outcomes of this process. In turn, this enabled cumulative cultural evolution and “cultural organisation in the form of the group’s specific set of conventions, norms and institutions” (Tomasello, 2018a, p. 666). These developments are described as enabled by a kind of psychology based on group-mindedness, defined by normative behaviour and group perspective taking (e.g., Tomasello et al., 2012; Tomasello, 2014, 2018a,b). According to SITh, both conventional- and moral norms constitute an evolutionary novelty that emerged in *Homo sapiens.* In this account, conventional norms refer to the right and wrong ways of performing instrumental activities, while moral norms refer to treating others right, i.e., in conformity with the sense of sympathy and fairness that was putatively inherited from *H. heidelbergensis* together with *joint intentionality*.

## 3. SITh through the lens of empirical evidence: recursive mind reading in apes and human children

According to SITh, recursive mind reading – the ability to represent embedded mental states (“we each know that the other knows etc.” Tomasello et al., 2012, p. 677) – is foundational and, thus, obligatory to SI. It is proclaimed to be “the basic cognitive ability that enables humans to engage in all forms of joint and collective intentionality (Tomasello, 2008, 2009), including joint attention, common conceptual ground, and all ‘public’ knowledge and activities” (Tomasello et al., 2012, p. 677). The two forms of ‘recursive mind reading’ that are most extensively discussed in SITh are joint attention and representing (i.e., holding knowledge) about others’ beliefs.

### 3.1. Joint attention

Joint attention is one of the most investigated topics in developmental science, where a broad spectrum of definitions and behavioural markers have been proposed (for recent overviews see Bard et al., 2021; Graham et al., 2021), all having in common the core of *triadic connectedness*, i.e., engaging with one or more social partners about a shared topic (e.g., Bard et al., 2021). In the field of comparative cognition, research on joint attention is dominated by the SITh perspective, where joint attention is theorised as the ability to *purposefully* coordinate attention with another individual *with the cooperative motivation* of sharing focus of attention and, thus, implicitly knowledge. Much of this research has focused on referential competence, with a strong bias toward investigating pointing comprehension and production in variants of the object-choice task. For example, a popular setup for testing pointing comprehension (as a way of probing *receptive* joint attention) implicates a (typically human) experimenter pointing to one of two containers to indicate the location of a reward. The SITh literature commonly reports that, in this type of setup, human infants succeed at choosing the correct (i.e., the indicated) container while apes do not (reviewed in Tomasello and Carpenter, 2005; Tomasello et al., 2005). Successful performance in this setup is interpreted as entailing that “the recipient *infers* that the communicator *intends* that she *know* that the food is in the bucket” (Tomasello, 2014, p. 57). Overall, it is thus concluded that human infants – but not apes – exhibit an understanding of cooperative intentions through information sharing (Tomasello and Carpenter, 2005; Tomasello et al., 2005, 2012).

The empirical record, however, does not support this conclusion, given evidence that, in certain situations, apes’ performance in the object-choice task conforms to SITh’s behavioural criteria for receptive joint attention (Itakura et al., 1999; Lyn et al., 2010; Leavens et al., 2019; Hopkins et al., 2022, *this Frontiers Research Topic*). Overall, the empirical record reveals significant individual variability among tested apes, and points to developmental, methodological and biological factors that may account for success or failure in understanding indications in the object-choice task. As such, apes that had more extensive exposure to human communication and social interaction during the rearing period (so-called ‘enculturated’ apes), exhibit better performance in object-choice tests that use pointing as the sole referential cue (for reviews see Leavens, 2011; Krause et al., 2018; Leavens et al., 2019; Bard et al., 2021). There is also data showing that point-following may be quickly learned by non-enculturated apes (e.g., Itakura et al., 1999). Moreover, both enculturated and non-enculturated apes perform better in tasks deploying more ecologically valid referential cues, such as vocalising or orienting gaze and body toward the referred object (Itakura et al., 1999; Lyn et al., 2010). Finally, with respect to underlying biological factors, successful performance in the object-choice task by chimpanzees is predicted by grey matter volume in brain structures within the posterior attention system (Hopkins et al., 2022), which has been proposed to support receptive joint attention in humans (Mundy and Newell, 2007).

Variants of the object-choice task have also been used for testing pointing production as a way of probing *initiation* of joint attention. A distinction is often made in this context between *imperative* and *declarative* pointing, which are meant to reflect distinct underlying intentions (for reviews and discussions see Tomasello et al., 2005; Leavens et al., 2019; Bard et al., 2021). As such, *imperative* pointing is described as aimed at eliciting an action from the interaction partner that will benefit the pointing individual, while *declarative* pointing would reflect a motivation to share information with another individual and, thus, ‘true’ joint attention and SI. Generally, SITh reports either that apes do not point for others at all, or that apes exhibit only *imperative* pointing (or other indication behaviours), thereby promoting the view that apes indicate for others to fulfil their own individualistic goals but not to altruistically inform others (e.g., Carpenter et al., 1998; Liszkowski et al., 2004, 2006; Tomasello et al., 2012, etc.).

It is worth pointing out, however, that much of the reasoning above is the outcome of differential conceptualisation rather than differential behavioural manifestation. When the imperative-declarative classification was originally proposed (Bates et al., 1975), both types were conceived as requests (or instrumental acts) which differed only with respect to their target, i.e., whether the request was for an object or the attention of the interaction partner. The potential of reflecting ‘individualistic’ goals is found in both types of pointing – or more correctly put, contexts, considering that the putative instrumental or cooperative intentions of indication behaviours are assessed based on context (e.g., design features of experimental tasks), rather than specific behavioural markers. Context manipulation, however, may be an insufficient criterion, given the absence of unequivocal, independent ways to measure that distinct task demands obligatorily and singly elicit certain intention types.

These operational limitations notwithstanding, the empirical record shows that, in certain circumstances, apes exhibit pointing, including pointing that can be interpreted as *declarative*. Pointing has been reported in interactions with both humans and conspecifics, in both enculturated and non-enculturated apes, as well as in wild populations (see Leavens, 2011; Leavens et al., 2019 for recent reviews). While pointing frequency is low in non-enculturated apes, it nevertheless occurs, thus invalidating SITh claims that apes do not point ‘naturally’, and there is also the case that other indication behaviours may fulfil similar referential functions to pointing. For example, chimpanzees and bonobos are reported to use acoustically distinct vocalisations known as alert *hoos* to inform conspecifics about threats. Importantly, these vocalisations are used in a manner that is dependent on the knowledge status of the conspecifics, i.e., alert calls are more likely to be produced in the presence of an ignorant audience (Crockford et al., 2012; Girard-Buttoz et al., 2020). Thus, these vocalisations appear to be deployed with a *cooperative* intention, i.e., to manipulate the conspecifics’ behaviour to their own benefit rather than to the benefit of the caller (Those who argue that chimpanzees are unable to vocalise intentionally still have to explain why the knowledge status of the conspecifics affect the vocalising behaviour in the direction of helpfulness).

Conversely, data from cross-cultural studies in our own species show that manual pointing is not the universally preferred referential behaviour of humankind, and that alternative referential behaviours are prevalent in some cultures (Cooperrider et al., 2018 and references therein). In fact, a recent study that examined joint attention (conceptualised as triadic connectedness) in samples of human and chimpanzee infants raised in six distinct socio-ecological settings (three distinct settings for each species), failed to find any human-specific characteristic of it (Bard et al., 2021). This study reported significant within-species differences with respect to the behavioural markers and contextual parameters that characterise triadic connectedness in humans, thereby demonstrating that the narrow and cognitively demanding definition of joint attention advanced by SITh is not representative of humankind. Within-species diversity was also found in chimpanzee infants, and the range of variation found in human infants overlapped with – rather than being distinct from – that found in chimpanzee infants.

Finally, another SITh argument for the absence of joint attention in apes is the claim that, unlike children, apes do not exhibit positive affect and gaze alternation between the focal object (or event) of joint action and their interaction partners during episodes of triadic connectedness. Such behaviours have been described as specific indicators of the ‘jointness’ of joint attention (e.g., Liszkowski et al., 2004; Carpenter and Call, 2013). The currently available empirical record contradicts this claim in at least three ways. First, as demonstrated by Bard et al. (2021), positive affect during episodes of triadic connectedness is a criterion that lacks universality in the human species. While it may be highly frequent in WEIRD samples (i.e., samples from Western, Educated, Industrialised, Rich, and Democratic cultures), the same cannot be said by other socio-ecological settings. Second, naïve WEIRD observers cannot reliably distinguish looks with a ‘sharing’ function from looks with requestive functions in the context of (mother-infant) interaction from their own culture (Graham et al., 2021). Third, episodes of triadic connectedness may be accompanied by positive affect in both wild and captive chimpanzees at levels that are comparable to those found in human infants from certain socio-cultural groups (Bard et al., 2021).

Summing up, research on the comprehension and production of indication behaviours as a way of probing joint attention has yielded data that shows relative rather than absolute differences between commonly tested samples of captive apes and children socialised in WEIRD cultures. Moreover, there is significant within-species variability, with chimpanzees, bonobos, gorillas and orangutans socialised in WEIRD cultures performing more similarly to infants socialised in these cultures. In such ape populations, pointing emerges – just like in human children – spontaneously, i.e., through immersion in and interaction with a cultural context that exhibits pointing. As reviewed above, data from non-enculturated apes further shows that point-following may be learned quickly, after a dozen of exposures. Conversely, episodes of triadic connectedness sampled from non-WEIRD human cultures may not satisfy the SITh criteria for joint attention. In turn, this indicates that SITh criteria are not normative for *Homo sapiens* as a species. Instead, there is significant within-species variability with respect to behavioural markers deployed in triadic interactions, affective tone and the frequency and type of gaze behaviours accompanying such interactions. In comparative studies, there is important cross-task variability, whereby apes are more successful in tasks that rely on more ecologically valid indication behaviours, i.e., behaviours pertaining to the referential repertoire to which apes tend to be socialised when reared by / with conspecifics. Taken together, these findings refute the SITh claim that humans exhibit unique adaptations for referential communication (e.g., pointing) and, more broadly, joint attention.

### 3.2. The ability to represent others’ beliefs

The debate about the ability to represent others’ beliefs – the second form of ‘recursive mind reading’ that is more extensively discussed in SITh – revolves primarily around data from so-called ‘false-belief’ tests, which fall within three broad methodological paradigms: ‘classic’ tests, non-verbal active response tests, and ‘implicit’ tests. An oft-used classic test is the ‘Sally-Anne task’ which is a ‘change-of-location’ test in which a skit is enacted that introduces participants to two dolls called, e.g., Sally and Anne. Sally has a basket and Anne has a box. In the skit, Sally places a marble in her basket and then ‘goes for a walk’ (i.e., is moved out of participants’ sight). While Sally is away, Anne moves the marble to her own box. Children are then asked to indicate in which container will Sally think that she will find the marble, and the correct answer is, of course, the basket. In the other two paradigms participants are presented with similar scenarios as the above but are either required to produce an overt non-verbal response (e.g., fetching or moving an object; helping to open a box) or, as in implicit tests, to simply watch scenarios while their gaze behaviours are measured. Studies using the ‘violation-of-expectation’ approach measure looking times capitalising on infants’ tendency to look longer at scenarios that contradict their expectations. By the logic of this approach, false-belief detection would be indicated by longer looking times at the box, i.e., the ‘true’ location in the scenario above. Studies using the ‘anticipatory gaze’ approach record whether the participants direct their looks toward the location that reflects the actor’s false belief (for a recent review see Scott and Baillargeon, 2017). Depending on approach, human infants may exhibit performance consistent with false-belief attribution from 7-months (the violation-of-expectation approach) or 17-months of age (anticipatory gaze approach).

Initially, the view advanced by SITh (e.g., Tomasello et al., 2012; Tomasello, 2014) was that apes fail false-belief tests, which reflected the mixed – but primarily negative – evidence gathered at the time. For example, in a change-of-location test inspired by the Sally-Anne task, a ‘Communicator’ used a wooden block to mark one of two containers as containing food, to then either witness (true-belief condition) or not witness (false-belief condition) the transfer of the food into the other container (Call and Tomasello, 1999). To pass, the apes had to choose the food that contained the reward, i.e., in the true-belief condition they had to choose the marked container, while in the false-belief condition they had to choose the non-marked container. In the first study implementing this design, apes failed in the false-belief – but not in the true-belief – condition. Incidentally, this performance is intriguing since, by the criteria reviewed in Section 3.1, this behaviour would qualify as an understanding of cooperative intentions. However, in another study that used the same design, chimpanzees were reported to pass the false-belief condition but fail the true-belief condition (O’Connell and Dunbar, 2003).

Recent non-verbal studies that used simplified procedures inspired by the infant literature have convergently reported that apes pass false-belief tests. Accordingly, support for the presence of belief attribution in nonhuman primates comes from an active-response study showing that apes tailor their helping behaviour depending on the beliefs held by their interaction partners (Buttelmann et al., 2017). Moreover, an anticipatory gaze eye-tracking study showed that apes can predict others’ false-belief guided actions, as they looked in anticipation to the location where an actor believed to find a target object, rather than the location where the object was moved in the actor’s absence (Krupenye et al., 2016). Follow-up eye-tracking studies established that the apes’ anticipatory looks were driven by socio-cognitive mechanisms, rather than low-level associative cues (Krupenye et al., 2017) or simple rules that agents search for objects where they last have seen them (Kano et al., 2019). To discount the first alternative interpretation, Krupenye et al. (2017) demonstrated that apes do not exhibit anticipatory gazing when the false-belief scenarios are manipulated by removing the agents and replacing them with inanimate objects. To discount the second alternative interpretation, Kano et al. (2019) showed that the apes’ own experience with an opaque and a translucent barrier that looked similarly from afar, influenced their anticipatory gaze behaviours when viewing false-belief scenarios. Specifically, anticipatory gaze consistent with false-belief understanding was selectively present in the apes who had previously experienced the opaque (as opposed to the translucent) barrier rather than being indiscriminately deployed to the last location where the agent had last seen the target object. Interestingly, anticipatory gaze behaviours consistent with false-belief understanding have also been demonstrated in Japanese macaques (*Macaca fuscata*) in a study that also found that such responses are mediated by the medial prefrontal cortex (Hayashi et al., 2020).

To deal with the inconsistency between SITh claims and recent findings of false-belief understanding in nonhuman species, recent SITh versions argue that non-verbal false-belief tasks only require *individual* mental state reading. Infants and apes, it is argued, pass such tasks by deploying individualistic ape psychology, i.e., socio-cognitive abilities evolved for competing with others (Tomasello, 2018b). By contrast, classic false-belief tasks, which, according to SITh, can only be passed from 4 years of age, would implicate uniquely human skills and motivations of shared intentionality. Overall, the developmental trajectory of false-belief understanding is described in SITh as fitting a U-shaped curve, where 3-year-olds would exhibit a dip in performance. SITh uses this alleged U-shaped pattern to advance the claim that 4-year-olds approach the false-belief test by deploying the human *default* approach to social interaction, whereby they attempt “to *coordinate* their own and their social partner’s *differing, sometimes conflicting, perspectives*, often with an *objective perspective* also lurking in the background” (Tomasello, 2018b, p. 8492). A second claim derived from the alleged U-shaped curve is that the performance dip of 3-year-olds reflects a developmental transition that parallels the evolutionary transition to *collective intentionality* (summarised in Section 2). Specifically, at 3 years of age children would experience the emergence of objective perspective-taking, social emotions, normativity and group-mindedness. This developmental transition would thus be responsible for the 3-year-olds’ failure in false-belief tests, since 3-year-olds would not be able to stand the ‘pull of the real’, i.e., they would not be able to control the prepotent salience of what is objectively true (Tomasello, 2018b). Moreover, since objective perspective-taking is an ability that is believed to not yet be stabilised at this age, 3-year-olds would tend to over-apply it, “assuming that people guide their search for things by an objective perspective” (Tomasello, 2018b).

There are, however, reasons to question this updated SITh interpretation of the performance of infants and apes in non-verbal false-belief tasks. The claim that apes and infants approach false-belief tasks with individualistic (rather than cooperative) intentions is inconsistent with the findings of Buttelmann et al. (2009, 2017), considering that the task used in these studies required participants (children and apes, respectively) to deploy a *cooperative* response that was dependent on the participants’ understanding of false-belief. Moreover, the U-shaped developmental pattern described by SITh does not seem to be supported by empirical evidence. Research shows instead that there is an incremental progression in both true and false belief understanding from infancy to the school years. Moreover, the claim that 3-year-olds underperform due to a putative ‘pull of the real’ is inconsistent with several empirical findings. First, the ‘pull of the real’ likely has no effect on 3-year-olds since they are successful in non-verbal false-belief tests and perform at levels comparable to those exhibited by 4-year-olds (Grosse Wiesmann et al., 2017). Second, the performance of toddlers and pre-schoolers is similar to that of human adults in non-verbal tasks that measure gaze behaviours (Wang and Leslie, 2016). Third, spontaneous reasoning about false- and true beliefs has been reported for both toddlers and 3-year-olds (Garnham and Perner, 2001; He et al., 2012; Moll et al., 2016). Fourth, 3-year-olds and even toddlers can succeed in classic false-belief tasks when non-essential, yet disrupting, task elements are eliminated (Carpenter et al., 2002; Rubio-Fernández and Geurts, 2013; Setoh et al., 2016). Overall, this body of findings suggests that true- and false-belief understanding emerge simultaneously rather than sequentially and develop gradually from infancy to the school years.

The developmental pattern used by SITh as an argument for the evolutionary outline of SI is also inconsistent with data from cross-cultural research. This research shows that the developmental trajectory of belief understanding exhibits cultural specificity both with respect to its onset and the sequencing of relevant ‘mind reading’ abilities. As such, children from a range of non-WEIRD cultures (e.g., China: Liu et al., 2008; Japan: Naito and Koyama, 2006; Pakistan: Nawaz et al., 2015; the Philippines: De Gracia et al., 2016; Samoa: Mayer and Träuble, 2015; Vanuatu: Dixson et al., 2018) appear to exhibit a developmental delay of up to 4 years compared to WEIRD samples (for reviews and metanalyses see Wellman et al., 2001; Liu et al., 2008; Heyes and Frith, 2014; Slaughter and Perez-Zapata, 2014; Aival-Naveh et al., 2019). This evidence shows, moreover, that WEIRD individualistic cultural settings promote the emergence of the understanding that people may hold different beliefs prior to the understanding that seeing entails knowing, whereas non-WEIRD settings exhibit the opposite pattern (for reviews, see Wellman et al., 2001; Liu et al., 2008; Heyes and Frith, 2014; Slaughter and Perez-Zapata, 2014; Aival-Naveh et al., 2019).

Intriguingly, cross-cultural differences appear to only affect children’s performance in classic (‘explicit’) tests of false-belief understanding. Conversely, studies using non-verbal false-belief tests report cross-cultural similarity (e.g., Moriguchi et al., 2010; Wang et al., 2012; Barrett et al., 2013). This performance pattern highlights yet another inconsistency with SITh claims, in that *only* the successful performance in non-verbal tests seems to point to an ability that could potentially be considered an adaptation, given early ontogenetic emergence and apparent cross-cultural universality [see also Barrett et al. (2013) for a similar point]. In contrast, successful performance in classic tests is characterised by cross-culturally variable onset and developmental sequence with respect to other ‘mind reading’ abilities, which is consistent with a socio-ecologically shaped ability. A range of socio-cultural factors have been discussed as mediating the cross-cultural variability of performance in classic tests, including language factors (e.g., the richness of the mentalising vocabulary), parenting style, individualistic (autonomy-valuing) vs. collectivistic (interdependence-valuing) social orientation, and analytic vs. holistic cognitive style (for reviews, see Wellman et al., 2001; Liu et al., 2008; Heyes and Frith, 2014; Slaughter and Perez-Zapata, 2014; Aival-Naveh et al., 2019).

Summing up, SITh claims about the evolution of SI and human-specific cooperation are based on a specific developmental trajectory of relevant socio-cognitive abilities. Among these claims is the contention that successful performance in implicit false-belief tasks merely requires individualistic ape psychology whereas successful performance in classic tests requires human cooperative psychology. However, the comparative, developmental and cross-cultural evidence reviewed in this section undermines the developmental outline proposed by SITh, thereby also undermining the evolutionary claims of SITh. The pattern that emerges is that non-verbal false-belief tasks that reveal cross-cultural similarity also reveal cross-species continuity. By contrast, performance in tasks invoked to argue for cross-species differences also exhibits considerable cross-cultural discontinuity. It is worth noting, however, that both SITh-promoted and alternative interpretations are currently affected by the limited comparability of the available data from implicit false-belief studies. First, as reviewed in this section, the nonhuman primate data come exclusively from anticipatory gaze studies. Second, in research with human infants, violation-of-expectation appears to be more frequently used and also a more reliable approach than anticipatory gaze (Barone et al., 2019). Third, the anticipatory gaze approach is prevalent in cross-cultural studies with pre-schoolers (Moriguchi et al., 2010; Wang et al., 2012; Barrett et al., 2013) and in within-species comparisons between, e.g., human children and adults (Wang and Leslie, 2016). Given this asymmetric distribution of the two main implicit approaches among the studied populations, the ape data appears more comparable to the human pre-schooler and adult data, than to the human infant data.

## 4. The prosociality cluster: altruism and cooperation

As reviewed above, SITh postulates that a radical divide separates ape psychology from human psychology. While apes are claimed to rely on an individualistic psychology encompassing skills and motivations evolved for competition, humans are claimed to have uniquely evolved an altruistic psychology that encompasses skills evolved for cooperation (Tomasello et al., 2012; Tomasello, 2018a,b, 2022). Arguments pertaining to the pro-sociality cluster invoked to support this central claim of SITh include claims that apes do not help others to the same extent as human children, do not exhibit mutual helping nor proactive helping, do not collaborate or prefer solitary as opposed to cooperative foraging, do not (equitably) share spoils resulting from dyad/group foraging, do not benefit others’ goals by sharing information and do not coordinate through communication in collaborative tasks. As per SITh, such prosocial behaviours and the mechanisms that reinforce them (e.g., partner choice, partner control) have emerged with *Homo heidelbergensis*, during the *joint intentionality* phase of SITh. In what follows, we take a closer look at SITh claims and (counter)evidence, progressing from studies on instrumental helping, to studies on resource sharing and cooperation, to conclude with studies on cooperative communication.

### 4.1. Instrumental helping

In SITh, helping behaviour is postulated to be a consequence of obligate cooperative foraging, which “produces interdependence among members of a group, and this interdependence makes it in my direct interest to help others who might be my future partners” (Tomasello et al., 2012, p. 679). As usual, SITh arguments are built around performance contrasts between human (WEIRD) children and apes. With respect to instrumental helping, the developmental data cited in SITh indicate that, at 14 months of age, human children help others retrieve desired objects (Warneken and Tomasello, 2007). By 18–20 months of age, they help others to achieve a wider array of more complex goals, such as opening a cabinet door (Warneken and Tomasello, 2006), and they do so even when it entails a cost such as locomoting some distance (Warneken et al., 2007; Warneken and Tomasello, 2009) or interrupting a desirable activity (Warneken and Tomasello, 2009). Interestingly, the performance of chimpanzees tested in similar experimental settings parallels that of children. Both human-raised and wild-born chimpanzees spontaneously help others retrieve desired objects in the absence of any reward and even when there are costs entailed by locomoting some distance (Warneken and Tomasello, 2006; Warneken et al., 2007). Moreover, chimpanzees have been reported to help conspecifics access food (Warneken et al., 2007; Melis et al., 2008) or non-food items (Melis et al., 2011) by unlocking a door or providing them with a needed tool (Yamamoto et al., 2009).

Overall, based on the findings reviewed above, SITh advances the conclusion that owing to their altruistic psychology “humans would seem to do it [i.e. help] much more frequently and in a much wider array of contexts, including actively sharing resources and information more freely (i.e., informing others of things helpfully and even teaching them things)” (Tomasello et al., 2012, p. 679). As pointed out in SITh, however, chimpanzees helping appears to be dependent on overt requests from the individual in need of help (Melis et al., 2011). This has been taken as an indication that human infants present unique adaptations geared toward cooperation, such as enhanced capacities to read signals of need, which enable them to engage in proactive helping. It is important to point out, however, that there is a discrepancy between the body of findings reviewed in SITh (and above) and the conclusion drawn from them. The empirical data clearly demonstrates that chimpanzees exhibit helping behaviours in a range of contexts that exceeds that found in toddlers, and which is comparable to that found in pre-schoolers. Overall, this suggests once more that the posited divide between ape and human psychology is instead a continuum, as altruistic tendencies are clearly demonstrated by chimpanzees in a helping context. Let us reiterate at this point that this conclusion should not be understood as a claim that chimpanzee and human forms of altruism and cooperation have not diverged considerably since the last common ancestor. It is obvious that they have. What we criticise is the characterization of the respective species in the SITh framework that has resulted from a selective representation of research, which risks simplifying the phenomena under study and narrow the focus of future research.

### 4.2. Resource sharing and cooperation

Field reports have revealed decades ago that chimpanzees engage in a range of prosocial behaviours beyond helping, including food sharing (de Waal, 2008), third-party consolation (Kutsukake and Castles, 2004; Romero et al., 2010), infant adoption (Boesch et al., 2010; Hobaiter et al., 2014), as well as multi-party collaborative activities, such as monkey hunting, territorial patrolling, coalitionary support, predator mobbing, and coalitionary mate guarding (for reviews see, e.g., Muller and Mitani, 2005; Boehm, 2018). According to SITh, however, such activities bear only a superficial resemblance to cooperation, being, in fact, driven by the individualistic goals of the participants as opposed to altruistic and shared group goals (Tomasello et al., 2012; Tomasello, 2018a). To support this interpretation, SITh cites two types of data. The first comes from experimental studies on resource donation (e.g., *prosocial choice, costly choice,* etc.) in which the performance of (WEIRD) children appears to be qualitatively different from that of captive apes, seemingly revealing human-unique features of altruism. The second type of data comes from studies of food sharing in the wild, but selectively comprises only those analyses which suggest that food sharing may be the result of non-prosocial factors such as harassment or proximity to the captor (Stevens and Stephens, 2002; Gilby, 2006).

#### 4.2.1. Resource sharing experiments

In a *prosocial choice* paradigm frequently mentioned in SITh, participants are given the option of manipulating one of two baited boards: a board that results in a food reward for themselves (*selfish choice*) and a board that rewards both themselves and a conspecific *(prosocial choice).* In such studies, chimpanzees appear to not discriminate between the two options, by choosing randomly or making *prosocial choices* in the absence of a conspecific (Silk et al., 2005; Jensen et al., 2006; Vonk et al., 2008; Yamamoto and Tanaka, 2010; Amici et al., 2014). In contrast, young children are reported to favour the option that benefits both themselves and an interaction partner and an oft-cited study in this respect is Brownell et al. (2009). Based on these reports, the conclusion advanced by SITh is that chimpanzees are insensitive to the welfare of others and have only limited sensitivity to their needs.

At closer scrutiny, however, the more complete developmental picture is that 18-month-olds chose randomly, and 2-year-olds chose prosocially *only* if the bystander verbalises her desire for the food – a behaviour that could also be interpreted as complying with a request made by an adult. By comparison, in the chimpanzee study, the bystander conspecific has not been reported to vocalise or exhibit other attention-getting behaviours. Since children chose prosocially only in a condition that was not testable in chimpanzees, the interpretation that, unlike chimpanzees, children behave prosocially in this paradigm is moot. Importantly, the broader developmental evidence from studies applying this paradigm is mixed. Some studies report, for example, that 4- and even 5-year-old children fail to exhibit prosocial responses, and that pro-social choice is manifested first at 7 years of age (Fehr et al., 2008; Claidière et al., 2015). In another study that tested children of various ages (3–4, 5–6, and 7–8 years), the children chose the prosocial board in 59% of the trials when another child was present, and 50% of the time when another child was absent (House et al., 2012), while children aged 5–6 failed to exhibit even such meagre levels of prosocial choice (a failure which the authors attributed to joking attempts by children of this age group).

It is also important to note that the chimpanzee studies invoked in support of SITh present several methodological concerns. For example, both Jensen et al. (2006) and Vonk et al. (2008) reported a side bias, which sometimes is an indication that the task contingencies are not understood by the subjects. Such side biases likely accounted for the random choice patterns reported in these studies, since choice options are typically counterbalanced with respect to side. In Jensen et al. (2006), for example, the chimpanzees chose predominantly the board that made the food accessible to the conspecific, but they did so in the control condition as well, i.e., when the conspecific was absent from the adjacent room. The authors comment themselves that, overall, chimpanzees did not emerge as selfish, since they did not try to keep food away from the bystander conspecific. Moreover, the task used by Vonk et al. (2008) relied on differential responses – push away for prosocial choice, pull toward self for selfish choice – which disadvantaged the expression of prosociality considering that chimpanzees (as well as bonobos, gorillas and orangutans) have a strong bias against pushing a food item away from themselves (Girndt et al., 2008). Finally, communication, which has been found to increase prosocial choice in children, was inhibited in these studies, as the chimpanzees were situated in different rooms, some 3 meters away and separated by double barriers.

Proving the point above, alternative paradigms designed to address methodological complexity and confounds have produced alternative findings (e.g., Horner et al., 2011; House et al., 2014), which invalidate SITh arguments that apes do not exhibit prosocial choices. For example, rather than using a complex apparatus, Horner et al. (2011) presented chimpanzees with a bucket containing two types of tokens: a type that rewarded the subject only and a type that rewarded both the subject and a conspecific. In this setup, the chimpanzees overwhelmingly favoured the prosocial option, but only when the bystander was present. The distance between the subject and the bystander was also decreased by having the two participants located in adjacent rooms, which encouraged communication. The results showed that attention-getting behaviours promoted subsequent prosocial choice, similarly to the results with children (Brownell et al., 2009) reviewed above. Importantly, only begging behaviour had this effect, while harassment inhibited prosocial choices. This is inconsistent with SITh claims that chimpanzee food sharing, rather than being the expression of altruistic motivations, is the result of giving up to harassment by conspecifics.

Similar findings come from a more recent study by Claidière et al. (2015) who administered a modified version of the two-board paradigm to human children and human adults, as well as to chimpanzees and capuchin monkeys. In this modified version, the distance between subject and bystander was minimised for all samples, and additional conditions were introduced by varying the type of reward – more preferred vs. less preferred - received by the subject and the bystander. Claidière et al. (2015) also added a subsequent test-phase in order to test if prosocial behaviour could be shaped and, indeed, promoted by prior experience with being the target of prosocial behaviours. In this study, 5-year-old human children did not exhibit prosocial choices, while 7-year-olds did so but only in the condition in which they received the more preferred reward. The capuchin monkeys performed like the young children, while the chimpanzees exhibited conditional prosocial choice similar to that of older children. However, when the salience of the reward was minimised (by delaying the receipt of the reward relative to the moment of choice), also capuchin monkeys exhibited prosocial choices. In contrast to all the samples, human adults exhibited unconditional prosocial responses. Interestingly, both chimpanzees and 7-year-old children – but not 5-year-old children – exhibited higher levels of prosocial choice after experiencing others’ prosocial choices toward themselves. Overall, and contrary to SITh, these findings point to a slow developmental trajectory of prosocial choices in human children and suggest that prosocial tendencies can be easily influenced by social learning (and thus cultural shaping) in school children and chimpanzees, thereby enabling generalised (rather than merely direct) reciprocity.

An even stronger expression of altruism is captured by studies on *costly prosocial choices*, where individuals benefit others at no gain for themselves, or even by incurring a personal cost. Such costly choices appear to emerge in human children at 4–5 years of age, although at this age they are biased toward friends (Moore, 2009). Based on SITh claims (as reviewed in the introduction of this section), this aspect of altruism would be absent in apes, but recent studies point to the contrary (Schmelz et al., 2017; van Leeuwen et al., 2021). van Leeuwen et al. (2021) allowed chimpanzee to freely engage with a task where button presses provisioned conspecifics – but not the button pusher – with juice from a fountain located at some distance (van Leeuwen et al., 2021). As the study progressed, the chimpanzees increased both the frequency and the duration of button presses. Shorter and less frequently presses were, by contrast, registered when button presses produced juice from a fountain outside the enclosure which, thus, did not benefit conspecifics. This pattern of results suggests that the chimpanzees purposely pushed the button to benefit group members in the absence of personal gain. Importantly, van Leeuwen and colleagues tested 3 chimpanzee groups from the same sanctuary and found significant between-group differences in prosocial behaviour, that were predicted by social variables. Specifically, levels of social tolerance (measured as the presence of aggression) positively predicted prosociality, while group size did not yield a significant effect. Moreover, kinship positively predicted prosociality only in the low-tolerance group, while in the high-tolerance groups prosocial clusters included chimpanzees that were not kin-related.

In the study of Schmelz et al. (2017), chimpanzees were found to prefer giving up 25% of their payoff to a conspecific rather than choosing 100% payoff for themselves. Such costly choices were found to be conditional on two aspects of prior interactional history with the conspecifics in question: whether the conspecific had shown prior prosocial choice toward the subject, and whether the conspecifics incurred the risk of food loss in prior encounters with the subject. Thus, similarly to the findings of Claidière et al. (2015), experiencing prosocial behaviour promoted prosocial responses, but in Schmelz et al. (2017) this took the form of *direct* reciprocity as opposed to the *generalised* reciprocity documented by Claidière et al. (2015). The chimpanzees specifically benefited those who had benefited them in previous interactions, rather than generalising prosocial behaviour irrespective of recipient. Such short-term reciprocation patterns were found to be independent of dominance rank or pre-existing social relationships.

#### 4.2.2. Observations and naturalistic experiments on food sharing

While the evidence of short-term reciprocation in apes is currently scarce, there is considerable data for medium- and long-term reciprocation involving food sharing, as well as the exchange of other benefits, such as grooming, coalitionary support, etc. Food sharing, especially when resulting from collaborative effort, has long been considered a human-unique trait [as discussed, e.g., by de Waal (1989)], of key interest for understanding the origins of human cooperation (Dart, 1953; Isaac, 1978; Lovejoy, 1981; Gurven and Hill, 2009; Jaeggi and Gurven, 2013a). Currently, evidence of food sharing has been documented in several mammalian and avian taxa where it is, however, limited to the mother-offspring context (for a concise overview see, e.g., Kaufhold and Rossano, 2020). Food sharing between adults, which, by contrast, is considered to be a rare trait in nonhuman animals, has been documented in all ape species, albeit with species-specific characteristics (chimpanzees: Goodall, 1963; Teleki, 1973; de Waal, 1989, 1997; Nishida et al., 1992; Boesch, 1994; Mitani and Watts, 2001; Watts and Mitani, 2002; Slocombe and Newton-Fisher, 2005; Hockings et al., 2007; Gomes and Boesch, 2009; Crick et al., 2013; Eppley et al., 2013; Silk et al., 2013; Wittig et al., 2014; Calcutt et al., 2014; bonobos: (Kano, 1980; Kuroda, 1984; Hohmann and Fruth, 2008; Surbeck et al., 2009; Hare and Kwetuenda, 2010; Tan and Hare, 2013; Yamamoto, 2015; Goldstone et al., 2016; Fruth and Hohmann, 2018; gorillas: Yamagiwa, 1992; Iwata, 2014; Yamamoto, 2015; orangutans: Bard, 1992; van Noordwijk and van Schaik, 2009; Kopp and Liebal, 2016; Kaufhold and Rossano, 2020).

Voluntary handing of food (so-called active sharing) is rare in apes, typically making up for 1–4% of all observed instances of food sharing by chimpanzees, bonobos or orangutans (Teleki, 1973; Kopp and Liebal, 2016), and, thus far, has not been reported in gorillas. The primary mode of food sharing is *passive,* whereby conspecifics are allowed to take others’ food without resistance from the possessor. It is commonly argued that such food sharing requires considerable levels of inhibitory control over potential aggressive responses when food is removed from one’s possession (Jaeggi and Gurven, 2013b). *Active* food sharing has been hypothesised to also require the ability to recognise and respond to the needs of others (idem) in addition to inhibitory control over aggression. It is, however, not excluded that recognising others’ needs is also implicated in passive food sharing, as begging seems to play a stimulating role in food sharing, as will be detailed further below.

In chimpanzees, the most well-studied food sharing behaviour is the sharing of meat obtained through group hunting by parties typically composed of males. The frequency of capturing animal prey is variable across sites and chimpanzee communities, ranging from less than once a month at Bossou (Guinea) to 10 times a month in the Taï forest (Ivory Coast), but generally nearly all meat hunts result in sharing. Several hypotheses have been proposed to account for the ultimate and proximate functions of this seemingly altruistic behaviour. According to the ‘sharing-under-pressure’ hypothesis, which is favoured by SITh (e.g., Tomasello et al., 2012; Tomasello, 2018a), chimpanzees share to mitigate harassment by conspecifics, as defending highly desirable resources would be costlier than sharing (Gilby, 2006). This interpretation is consistent with some reports of high aggression levels and persistent harassment during meat sharing by the chimpanzees at Gombe (Gilby, 2006). Alternatively, since hunting is energetically costly and potentially risky, meat possession and sharing was suggested to be a form of (costly) signalling (Gombe – Tutin, 1979) or a way of increasing social status (Teleki, 1973; Boesch, 1994).

Currently, however, strongest empirical support is garnered by the ‘reciprocity’ hypothesis, whereby meat sharing is used in exchange for social ‘commodities’, such as grooming, access to mates, forging and maintaining alliances or affiliative relationships (for reviews see Jaeggi and Gurven, 2013a,b). Among studied populations there is considerable variation with respect to exchanged currency and food flow, even though, in wild populations, food donors are (almost) exclusively males. For example, in a chimpanzee community from Mahale, the food flow was found to predominantly go from the alpha male to its closest allies, thereby suggesting meat sharing as a coalition strategy (Nishida et al., 1992). Reports from the Taï forest reveal preferential meat sharing among hunt participants, irrespective of pre-existing relations, thereby suggesting a more equitable sharing strategy where meat rewards cooperative efforts (Boesch, 1994; Samuni et al., 2018). Other studies have reported meat flow from males to (oestrous) females, thereby suggesting meat sharing as a strategy to secure access to oestrous females or to strengthen intersexual relationships for increasing future mating opportunities (Boesch, 1994; Gomes and Boesch, 2009). Finally, meat sharing may be a strategy to strengthen social bonds (Wittig et al., 2014), a function that has also been suggested for the sharing of less valuable (and more widely accessible) plant foods (Slocombe and Newton-Fisher, 2005).

The sharing of plant foods is infrequently reported by field studies on chimpanzees, but virtually all studies on food sharing in captive populations use plant foods. In the wild, the chimpanzees from Bossou (Guinea) have been reported to share valuable crop-raided foods. Just like meat hunting, crop-raiding is a risky activity, especially considering that the raiding parties preferentially raid in the presence of humans and in highly exposed locations (Hockings et al., 2007). Compared to meat sharing, raided crops are less frequently shared, i.e., in up to 10% of raids compared to nearly all hunts. While hunting for meat typically takes place within a group’s territory, crop raiding entails spatial and temporal displacement, as chimpanzees raid in nearby villages and, when sharing, they transport raided foods from the village to their ‘home base’ in the forest. With respect to food flow, raided crops are transferred by males primarily to females of reproductive age and, in some instances, to their mothers, while females have never been observed to share with unrelated adults. Females, though, have been observed to share meat with others – although in this community, given the kind of species available, catching animal prey is a low-risk activity that is carried out individually. Based on the currently limited data, several functional hypotheses are simultaneously consistent with raided-crop sharing, including the costly signalling hypothesis, the food-for-sex hypothesis, and the food-for-grooming hypothesis (Hockings et al., 2007).

Compared to field studies, research with captive populations reveals both similarities and differences with respect to exchanged currencies and food flow. A significant difference is that food transfer by females is infrequently reported by field studies, but occurs with high frequency in captive populations, where both low and high value foods may be shared (e.g., de Waal, 1989, 1997; Eppley et al., 2013; Calcutt et al., 2014). Among relevant similarities is that, paralleling the findings of field studies, research with captive chimpanzees reports significant correlations with respect to food sharing or between food sharing and social commodities (e.g., grooming, mating) thereby suggesting that bidirectional, reciprocal relationships underlie food transfer among adult chimpanzees, independent of kinship and rank. However, the studies conducted in captivity further extend the understanding of reciprocity gathered from wild populations, by reporting evidence of not only positive but also negative reciprocation, whereby individuals that exhibit low rates of food sharing are also more likely to be (harshly) refused when making food requests (e.g., de Waal, 1989). Moreover, it has been shown that the same individual will not extend two favours in a row to a conspecific (e.g., grooming followed later on by food sharing) thus leading to the suggestion that, in chimpanzees, “the exchange of social favours is guided by a turn-taking rule which prevents one-sided accumulation of benefits” (de Waal, 1989, p. 454).

Studies with captive populations have also refined the understanding of the motivational context of food sharing. Importantly, these studies underscore that competitive sharing involving forced claims by dominants, stealing and aggressive harassment – which is presented in SITh as characteristic of ape psychology (e.g., Tomasello et al., 2012; Tomasello, 2018a) – is rarely observed in adult chimpanzees, although aggressive tendencies and stealing may be heightened in immatures (de Waal, 1989). While dominant individuals may be found to have a higher degree of involvement in food sharing, this seems to be related to their more prominent role in the food flow as donors, not in terms of exclusive claims to resources, as suggested by SITh. Finally, studies with captive populations have generated data for assessing the ‘sharing-under-pressure’ hypothesis which states that food sharing by chimpanzees is a strategy to mitigate harassment. This research points to a clear distinction between begging behaviours and harassment. While the former tend to be more persistently exhibited by close affiliates and are conducive to food transfer, the latter is characterised by aggression and fails to elicit food sharing (Horner et al., 2011; Eppley et al., 2013). Interestingly, one study found that competition, monopolising and aggression remained low even when a highly valuable resource diminished (Calcutt et al., 2014). Diminished resources did not contribute to a decrease in time spent sharing, or to a decrease in the number of sharing individuals.

Although much more limited, the data on food sharing in wild bonobos indicate that, unlike wild chimpanzees, bonobos share primarily plant foods. Shared food items encompass both high value fruit, as well as lower value food, such as leaves, piths or fruits that are abundant and can easily be acquired by all members of the community (Kano, 1980; Kuroda, 1984; Fruth and Hohmann, 2002; Yamamoto, 2015; Goldstone et al., 2016). Meat sharing, which results from individual rather than group efforts, has also been reported (Fruth and Hohmann, 2002, 2018; Hohmann and Fruth, 2008; Surbeck et al., 2009), albeit at lower rates than in chimpanzees (e.g., twice/month at Lui Kotale in the Democratic Republic of Congo, based on Fruth and Hohmann, 2018). Moreover, among wild bonobos, food transfers flow primarily from females to other members of the community (Yamamoto, 2015).

Interestingly, food sharing has been observed to even occur between members of separate groups of bonobos (Tan and Hare, 2013; Tan et al., 2017; Fruth and Hohmann, 2018). Such findings go against SITh claims (e.g., Tomasello et al., 2012) that altruism (helping, sharing, cooperating, etc.) directed toward strangers is a human-specific trait. Fruth and Hohmann (2018), for example, described the transfer of antelope meat from a male captor to females from both his own and the neighbouring group. Cross-group sharing has been also reported by studies with captive bonobos (Tan and Hare, 2013; Tan et al., 2017), where it has been suggested to facilitate contact with strangers (Tan and Hare, 2013). This phenomenon has been attributed to higher levels of social tolerance toward neighbouring groups among bonobos when compared to chimpanzees (Tan and Hare, 2013; Fruth and Hohmann, 2018). It has been thus proposed that the evolution of prosociality toward unfamiliar individuals (xenophilia) may have evolved due to selection for social tolerance which enhanced the desirability of interacting with strangers and forging new relationships in extended social networks (Tan and Hare, 2013). While this tends to be a popular view, it is important to point out that the evidence concerning differences between chimpanzees and bonobos with respect to levels of social tolerance is mixed, given high variability across studied populations (Hare and Kwetuenda, 2010; Jaeggi et al., 2010; Bullinger et al., 2013; Tan and Hare, 2013).

With respect to functional hypotheses of food sharing by bonobos, the currently available data provides little support to the ‘reciprocity’ hypothesis, apart from more isolated observations of food sharing for sexual favours (Kuroda, 1984; Goldstone et al., 2016) and potential reciprocity of grooming-for-food in captivity (Jaeggi and Gurven, 2013a). The ‘sharing-under-pressure’ hypothesis is somewhat consistent with the food sharing patterns reported by Fruth and Hohmann (2002), whereby, among the bonobos of Lomako forest, the transfer of high-quality fruit and meat increased with the number of beggars. Other reports from the Wamba Forest and LuiKotale, however, fail to provide support to either of these hypotheses, favouring alternative interpretations such as ‘courtesy’ sharing (Yamamoto, 2015) or ‘information assessment’ sharing (Goldstone et al., 2016), whereby solicitors beg for foods as a way of probing (and increasing) the strength of social bonds. Overall, researchers agree that the currently available data is insufficient for drawing any firm conclusions. All proposed hypotheses may, in fact, receive support depending on contextual variables, such as type of shared food, seasonality, demographic features of specific populations, socio-ecological environment, etc. Study design may, furthermore, affect the outcome of data analysis. For example, studies may reach different conclusions about reciprocity depending on whether reciprocity patterns are evaluated in the short- or long-term (de Waal, 1989; Goldstone et al., 2016).

By comparison to chimpanzees and bonobos, the data on food sharing in gorillas is extremely scarce. In mountain gorillas *(Gorilla beringei beringei*), there are observations that older males frequently relinquish their feeding spots to younger ones following social staring used as a begging strategy (Yamagiwa, 1992). This phenomenon of feeding patch supplantation appears to concern only readily available food, and it has not (yet) been documented for seasonal fruit. According to recent reports, food sharing also occurs among the western lowland gorillas (*Gorilla gorilla gorilla*) of Moukalaba-Doudou Park (Gabon) in at least two contexts: food dropping (Iwata, 2014) and *Treculia* fruit sharing (Yamagiwa et al., 2014). In the food dropping context, individuals that forage in trees selectively drop edible items – primarily low-value leaves and sometimes fruit – to the ground when conspecifics are present below the trees but not when there are no potential recipients. The prevalent food flow appears to be from younger to older recipients, although individuals of all age and sex classes have been observed both as donors and recipients. Given the predominantly low value of the dropped food and the lack of aggression during this type of food transfer, the food dropping behaviour has been interpreted as a prosocial strategy to buffer against competition over food sites. *Treculia* fruit sharing exhibits similar food flow patterns, whereby all sex and age categories are involved as both donors and recipients except for infants, which are only involved as recipients (Yamagiwa et al., 2014). Given the involvement of a high-value food, however, as well as the absence of aggression and insistent begging, fruit sharing in gorillas appears consistent with hypotheses that highlight nutritional benefits to the recipients and social benefits to the donors, such as the reciprocity hypothesis and hypotheses centred around the probing and strengthening of social bonds.

Finally, food sharing beyond the mother-offspring context has also been reported in both Sumatran *(Pongo abelii)* and Borneo orangutans *(Pongo pygmeus)*. In the wild, food sharing seems restricted to an intersexual context, with unflanged males as prevalent donors and sexually active females as recipients (van Noordwijk and van Schaik, 2009). Successful food transfer from males to females, however, does not appear to promote sexual benefits in the short-term, but may function as a mechanism of female mate choice. Indeed, unsuccessful male-to-female food transfers were observed to elicit loud female protests, which, for the male entailed the termination of the association with the female. Food flow in the opposite direction, i.e., from females to males, was also opposed with loud protests. Most cases of intersexual food sharing involved food types of trivial value, that were readily available on site, and which females could possess just as frequently as males. Overall, these sharing patterns suggest potential sexual benefits for donors in the long-term (i.e., resulting from prolonging the association with a given female), and informational benefits for the recipients, in the form of assessing males’ tolerance levels, which in turn may be relevant for assessing the risk of violence in sexual interactions (van Noordwijk and van Schaik, 2009).

Food sharing in zoo housed orangutans presents remarkably different patterns compared to those summarised above. In two recent studies, the sharing of monopolisable food occurred frequently within several groups of Sumatran orangutans, ranging between 60 and 77% of all observed food interactions (Kopp and Liebal, 2016; Kaufhold and Rossano, 2020). Food transfers involved both male–female and female–female dyads, with relationship strength (independent of kinship) being positively associated with the likelihood of food sharing (Kopp and Liebal, 2016). In turn, this is consistent with long-term ‘reciprocity’ hypotheses, whereby the readiness to share food depends on partner value, which in turn is based on prior interactional history, including given, received and denied benefits (Jaeggi and Gurven, 2013a,b). In addition, Kaufhold and Rossano (2020) found evidence of food transfers influenced by short-term reciprocity, whereby food sharing was promoted by positive dyadic interactions prior to food availability.

Interestingly, Kopp and Liebal (2016) recorded a high proportion of *active* sharing (19%) in the studied populations, as well as sharing between individuals from neighbouring groups. Just like for the other ape species, harassing behaviours were virtually absent, but non-aggressive begging (peering, gestures) was frequent. Overall, the high-frequency and generality of food sharing among orangutans is puzzling given the semi-solitary lifestyle of orangutan species. Unlike all other hominid species, orangutans do not live in stable social groups. In the wild, related females (and their offspring) are occasionally co-located, which gives opportunities for a range of social interactions, which in rare occasions may include food sharing (van Noordwijk et al., 2012). By contrast, in captivity, orangutans are typically housed in groups, and affiliative behaviours between individuals are frequently observed. This is consistent with the hypothesis that the current social organisation of orangutans is a derived form of an ancestral state of group living, and potentially an adaptation to resource scarcity. Traits supporting higher levels of sociality than documented in wild populations, as well as prosociality – which characterise the *Hominidae* genus – are, thus, expected to have evolved in orangutan species.

#### 4.2.3. Mechanisms of food-related prosociality

Hominid food-related prosociality is supported by a robust repertoire of communicative signals related to foraging, which function as behavioural mechanisms for mitigating competition and potentially aggressive outcomes (e.g., de Waal, 1989; Yamagiwa, 1992; de Waal, 1997). As noted by several researchers, taking food from conspecifics, especially when these are larger, stronger, and more dominant poses risks of agonistic repercussions (e.g., de Waal, 1989; Yamagiwa, 1992; van Noordwijk and van Schaik, 2009; Jaeggi et al., 2010). Yet, among apes (as reviewed above), food flows frequently from high rank or stronger individuals to lower rank or weaker conspecifics, and aggression is uncommon. This suggests remarkable capabilities to inhibit aggressive tendencies in the food sharing context (de Waal, 1989; van Noordwijk and van Schaik, 2009), which are enhanced by tension-reducing behaviours. For example, bodily contact (e.g., embracing, kissing) is frequently observed among chimpanzees upon encountering resource-rich patches in the wild (Goodall, 1968), as well as prior to feeding in captivity (de Waal, 1989). In the same contexts, genito-genital contact is reported to increase among bonobos (Kuroda, 1984; Jaeggi et al., 2010). Prior to feeding, ‘rituals’ of hierarchy reinforcement, characterised by bluff displays and pant hoots by dominants and submissive behaviours by lower rank individuals, are common in chimpanzees. As argued by de Waal (1989, p. 452), this recurring suite of behaviours in a pre-feeding context may be regarded as “an unequivocal confirmation of the hierarchy just before the temporary suspension of priority rights in relation to food.”

The behavioural repertoire of food sharing further includes signals to share information about food (e.g., food grunts), as well as behaviours for requesting foods, such as peering/staring, tactile and visual gestures. Both bodily- and eye contact are known to stimulate the release of oxytocin, a neurohormone with anxiolytic effects that promotes affiliative relationships [for a recent review, see Jones et al. (2017)]. Oxytocin as an underlying neurochemical mechanisms of food sharing has been recently confirmed in chimpanzees, with oxytocin levels being higher after food sharing compared to grooming (Wittig et al., 2014). Increased oxytocin levels have also been reported after group hunting in chimpanzees (Samuni et al., 2018), as well as in non-foraging contexts of prosociality, such as intergroup agonism (Samuni et al., 2017).

The literature on food-sharing (reviewed above) and other forms of ape cooperation (e.g., intra- or intergroup agonism) points to a range of social selection mechanisms that mediate prosocial behaviour. Several of these mechanisms, including kin and affiliative relationships, partner choice based on skill and reciprocity, as well as partner control through punitive strategies, have been commonly discussed as competition-mitigating mechanisms central to the evolution of human altruism. SITh, in particular, makes the claim that social selection based on cooperative qualities emerged in *Homo heidelbergensis*, enabling SI in this species (e.g., Tomasello et al., 2012; Tomasello, 2018a). Experimental and observational data show that chimpanzees prefer to collaborate and share resources with kin, similar rank conspecifics or socially bonded individuals (e.g., Boesch, 1994; Eppley et al., 2013; Suchak et al., 2014; Kopp and Liebal, 2016; Samuni et al., 2021). However, preferential sharing and cooperation has also been reported toward individuals who are more skilled and effective cooperators (Melis et al., 2006a) or who have been prosocial in the recent past, thereby indicating the presence of short-term reciprocation of favours (Koyama et al., 2006; Melis et al., 2008; Schmelz et al., 2017; Kaufhold and Rossano, 2020) irrespective of pre-existing, long-term social relationships.

Partner control through punitive strategies has been documented in several forms in chimpanzees. For example, chimpanzees may reduce grooming individuals who did not offer expected conflict support (Koyama et al., 2006), exhibit delayed retaliation against individuals who aggressed them (de Waal and Luttrell, 1988), refuse food sharing with individuals that do not share (de Waal, 1989), or withhold engaging in cooperation in the presence of non-cooperators (Suchak et al., 2016). Attitude formation based on others’ pro- or antisocial behaviour, whether experienced directly or witnessed from a third-party perspective, has been reported in both chimpanzees and orangutans (Russell et al., 2008; Herrmann et al., 2013), leading to an avoidance of antisocial agents and a preference to interact with prosocial individuals. Chimpanzees and 6- (but not 4- and 5-) year-old children have been even reported to ‘pay’ (physical effort or monetary units, respectively) to be able to watch the punishment of antisocial individuals (Mendes et al., 2017). Finally, there are also reports of third-party punitive interventions against freeloaders, where dominant chimpanzees act against bystanders who attempt to access cooperatively obtained food without having engaged in cooperation (Suchak et al., 2016). Interestingly, evidence of third-party interventions has begun to accumulate for orangutans as well, although the extent to which this relates to unfair behaviour in the context of resource sharing remains to be established (Kopp and Liebal, 2018, and references therein).

The data reviewed above also contrast with SITh claims that only children – but not apes – are sensitive to equitable sharing of resources obtained through cooperative efforts (e.g., Tomasello et al., 2012). SITh cites findings that apes do not punish others in contexts when punitive responses would be expected due to unfair outcomes (Jensen et al., 2007; Riedl et al., 2012), and that they share at similar levels irrespective of how resources have been obtained (Melis et al., 2011), while 3-year-olds (Hamann et al., 2011), exhibit more equitable sharing when resources result from collaborative efforts. However, in the study of Melis et al. (2011), it was found that chimpanzees begged more in the cooperative condition, which, in turn, resulted in heightened sharing. While not significant, this difference needs to be weighed against findings from other studies indicating that begging (and thus sharing) in chimpanzees is mediated by pre-existing social relationships (Eppley et al., 2013). As mentioned above, begging is primarily exerted by social affiliates, which suggests that engineered dyads may yield biased experimental findings. Besides the evidence reviewed above which suggests third-party inequity aversion, there is additional evidence that chimpanzees may refuse to participate in a task based on inequity conditions, where they receive a lower payoff than a conspecific partner (e.g., Brosnan et al., 2010; for reviews, see Brosnan, 2011; Yamamoto and Takimoto, 2012). Such findings suggest once again that the evolution of human cooperation is better approached from a graded perspective rather than the dichotomous view advanced in SITh. Of relevance to the argument in focus, it has been argued that inequity aversion stabilises cooperation as a mechanism that keeps the balance between giving and receiving benefits, through two key mechanisms – partner choice and negative response to free-riders (e.g., Yamamoto and Takimoto, 2012). The findings reviewed above demonstrate the presence of such mechanisms in at least some ape populations and cooperative contexts and is consistent with the suggestion that “human forms of collaboration are built on a foundation of evolutionary precursors that are present in chimpanzees and a variety of other primate species” (Melis et al., 2006a, p. 1300).

#### 4.2.4. Within-species variability and methodological concerns

Overall, the data on prosocial choice and food sharing beyond the mother-offspring context reveal considerable behavioural diversity between studied populations and within each ape species. Within-species variability has been registered with respect to the frequency of sharing, types and value of shared foods, the presence of aggression around food sharing episodes, the patterns of food-flow, as well as the ultimate and/or proximate explanations that appear to be consistent with a given dataset. Notably, with respect to the latter aspect, the functional and/or mechanistic explanations proposed thus far are not mutually exclusive, and it is not infrequent that more than one explanation is consistent with a given dataset. Moreover, none of the explanations advanced so far has been unequivocally rejected, although, across studies, the hypothesis that gains least support is the ‘sharing-under-pressure’ hypothesis promoted in SITh. The diversity of functional and mechanistic accounts, as well as the substantial within-species differences documented in the studied populations (both from the wild and captivity), suggest high malleability of prosocial behaviours.

A certain amount of within-species variability can certainly be attributed to methodological differences. Sometimes, slight experimental changes may produce completely different results. For example, in a study commonly cited in SITh, chimpanzees preferred working on their own rather than cooperating with a conspecific when the reward was set at 2 banana pieces for each individual. However, they suddenly switched to an overwhelming preference for cooperation when the reward was increased to 3 pieces (Bullinger et al., 2011). In this study, working alone or with others did not entail different degrees of difficulty and obtaining the rewards was not contingent upon cooperation. In other studies, demographic variables may influence the outcome. For example, several studies reporting negative results on chimpanzee pro-sociality have drawn their data from samples of immatures (including the study of Bullinger et al., 2011 mentioned above) – a developmental stage that appears to be characterised by higher rates of aggression and competition around food (de Waal, 1989).

Of outmost significance with respect to methodological limitations is the discrepancy in reported outcomes between experimental data based on artificial setups, on the one hand, and field data, as well as experimental data based on naturalistic setups, on the other hand. As reviewed above, the former type of data (which is commonly invoked to support SITh) tends to yield negative results on the presence and range of prosocial behaviour in apes. Conversely, naturalistic designs are more likely to report positive evidence of ape prosociality. An important limitation of artificial setups is the use of engineered dyads, thereby disregarding the range of social variables that underlie cooperative behaviours in apes and, in fact, in humans as well. For example, higher levels of mutual tolerance may increase the likelihood of cooperation and sharing in an artificial setup, while cooperation is seen to break down in low-tolerance dyads (Melis et al., 2006b). In the same vein, when chimpanzees are allowed to freely choose cooperative partners, the frequency of cooperation is considerably increased to a point where chimpanzees cooperate continuously for as long as the apparatus is available (Suchak et al., 2014, 2016). Overall, the use of engineered dyads fails to consider the complex dynamics of apes’ social life, both in the long- and short-term, which, based on field observations, affects decisions when to cooperate, with whom to cooperate, and to whom refuse cooperation.

Other methodological advantages in naturalistic setups include prolonged study duration, as well as allowing the apes to freely explore and discover task contingencies. For example, in the study of Suchak et al. (2016), chimpanzees did not receive any pre-determined training on how to operate the apparatus. Instead, they learned by interacting with the apparatus and by experiencing the need to cooperate. The experiment was also a long-duration study that included 94 sessions. This enabled the researchers to discover that, after an initial increase in competition, there was a sharp rise in cooperation, which remained high for the remainder of the experiment. Similarly, in the juice provisioning study of van Leeuwen et al. (2021), behavioural choices that benefited recipients (but not the donor) increased as the study progressed. The two studies discussed here demonstrate that, when given enough time, chimpanzees may converge on a prosocial strategy when engaging with food-sharing tasks.

#### 4.2.5. The prosociality cluster: developmental and cross-cultural data

In advancing its claims that prosociality in the area of resource sharing is a human-specific adaptation, SITh rests heavily on developmental data. Beyond findings from WEIRD samples cited in SITh, however, the developmental evidence reveals once again a variability of outcomes both within and across human cultures. The experimental studies commonly cited by SITh seem to indicate an early emergence of spontaneous sharing and inequity aversion. By contrast, the broader developmental literature paints a highly equivocal picture of mixed results that, overall, tend to favour highly divergent developmental conclusions. For example, Rochat et al. (2009) administered a resource distribution task to 3- and 5-year-olds from 7 cultural groups, and found that the behaviour of young children was far from being as overwhelmingly generous as presented in SITh. When choosing between sharing a small number (6 or 7) of food items between themselves and the experimenter, in 4 of the 7 sampled cultures, children’s prevalent response was self-hoarding, i.e., keeping *all* the items to themselves. The results of Rochat et al. (2009) further showed that, in several but not all cultures, self-interest tended to decrease with age, but that, when higher-value items were at stake, in all (but one) cultures, and regardless of age, children were overwhelmingly selfish. A similar age shift was also documented in a sample of Tibetan children raised in an environment that strongly prescribes compassion for others: 3-year-olds exhibited a marked self-interest, while 5-year-olds showed more fairness (Robbins et al., 2016). As a side note, Rochat et al. (2009) also administered a false-belief test to their cross-cultural sample, given commonly held assumptions (also advanced by SITh) that prosocial behaviour – in particular, fairness – is related to the development of perspective taking and advanced mind-reading. This conjecture, however, was not supported, as successful performance in the false-belief test and the prosocial choice task were not positively associated in the studied populations.

In another seminal study (Birch and Billman, 1986), children’s tendency to share high- and low-value food was tested in a context that also varied the social relationship between possessor and recipient (friend, acquaintance), and assessed whether prior experience as a recipient influenced children’s prosocial responses. The study revealed that children (all enrolled in classes at the University of Illinois Child Development Laboratory) shared minimally, relinquishing in average 1.42 (out of 10) preferred food pieces toward friends, and 0.68 (out of 10 pieces) to acquaintances. With respect to non-preferred food items, children relinquished an average of 1.68 to friends, and 1.23 to acquaintances. The frequency of *active* sharing was very low (*M* = 0.8 food pieces shared with friends; *M* = 0.14 food pieces shared with acquaintances), and the predominant mode of sharing was *elicited* sharing (i.e., in response to requests: 1.44 pieces to friends, 0.72 pieces to acquaintances). Finally, the study found a gender X relationship interaction, where girls were more likely to share with friends (1.75 pieces) than acquaintances (0.75). In a more recent replication of this study with two cross-cultural samples from urban Hong Kong and Bangalore (Rao and Stewart, 1999), children exhibited relatively similar levels of high-value food transfer to friends (*M* = 1.31 in Hong Kong and *M* = 1.67 in Bangalore) but shared slightly more high-value food with acquaintances (1.58, 1.86 respectively) than the Illinois sample. Other cross-cultural differences were that the Hong Kong and the Bangalore samples transferred more low-value food to friends (2.19, 2.08 respectively) compared to the Illinois children, and children from Hong Kong in addition exhibited slightly higher transfer levels when sharing low-value food with acquaintances (1.97) compared to the Illinois children. With respect to modes of sharing, *active* sharing was higher among Hong Kong children (1.51) compared to both Illinois and Bangalore children (0.44), while *passive* sharing was significantly higher among Bangalore children (with an approximate average of 1).

While it can be argued that experimental settings present children with artificial situations that may affect their performance, young children are found to exhibit low levels of sharing in naturalistic contexts as well. For example, Eisenberg-Berg and Hand (1979) found an average of 2.7 prosocial incidents per hour in interactions with peers in school-aged children. More recently, Tavassoli et al. (2022) investigated the presence of prosocial response in collaborative contexts that confronted 3- and 6-year-olds from two cultural backgrounds (rural Mexico, urban Canada) with peers’ behavioural or verbal expressions of need. Overall, only 8.7% of expressed needs received a prosocial response, with 40% being explicitly denied a prosocial response and 50% being ignored. The findings of this study do not represent an isolated case, as similar levels of prosocial response to others in naturalistic settings is found by studies observing prosocial responses among siblings and friends (e.g., Tavassoli et al., 2019, 2020). This is inconsistent with SITh claims that humans have evolved species-unique adaptations to read the needs of others that emerge early in human infancy.

Overall, the developmental studies reviewed above tend to converge toward a developmental baseline of marked selfishness in early childhood. In several studies, levels of sharing exhibit lower levels than those reviewed for several ape species, while also suggesting – at least in some ape populations and samples of children – similar mediators of prosocial response, such as social bonds. Children’s fairness with respect to resource distribution, as well as other forms of prosociality, are characterised by significant variability, both within and across cultures, and variables such as resource desirability, sex, social bonding, and the presence and age of an interaction partner, may account for variable outcomes. The type of performance that SITh proposes as normative for human early childhood is far from being universally observed, thereby qualifying as one behavioural phenotype among others. In turn, what appears to be universal in human prosocial development is that a range of prosocial phenotypes develops through exposure to social norms that, across socio-ecological environments, also vary greatly with respect to their contents and prescriptive strength. The readers with parenthood experience (at least in WEIRD cultures) can surely testify that claims of possession over objects are a frequent cause of frustration and conflict in early childhood. However, starting with the age of entering preschool, children are intensively encouraged to relinquish possession through sharing (Tobin et al., 1989). This can also suggest that spontaneous prosociality in children may be driven by social desirability (and, thus, ultimately selfish) goals, rather than a motivation to benefit others.

Illustrating the point above, a recent cross-cultural study that sampled (up to) 8 cultural groups from 7 countries, confirmed that baseline levels of self-interest at 4 years of age are predominantly high, with moderate levels being the exception (House et al., 2020). This study further revealed cross-cultural differences in the developmental trajectory of prosocial choice, whereby levels of selfish choice in late childhood could be both higher and lower compared to baseline levels. Despite this diversity, House et al. (2020) uncovered a universal trait: in each studied culture there was a gradual compliance in levels of prosociality/selfishness with the social norms that governed sharing – and more broadly prosociality – in the given culture. Specifically, in late childhood (at about 10-years of age), children’s level of prosocial choices reliably resembled that exhibited by the adults of the respective cultural group. Notably, prosociality profiles expressed as the proportion of times adults chose to reward both themselves and another individual (as opposed to choosing a double reward for themselves) ranged from as low as 20% to as high as 78%, averaging 48% across the socio-cultural groups sampled by House et al. (2020).

While the findings above show that children’s *explicit* responsiveness to norms increases with age and exhibits a reliable effect on prosocial behaviour in late childhood, studies of *implicit* expectations suggest that socio-cultural forces act on shaping prosociality from very early on. For example, a recent looking-time study found that infants (aged 12–21 months) from distinct socio-cultural backgrounds (urban Gothenburg in Sweden, Kikuyu children from rural households in Laikipia East in Central Kenya, and Samburu children from rural settlements in Wamba East in Northern and Central Kenya) respond differentially when presented with egalitarian and non-egalitarian sharing scenarios, and in a manner that is consistent with their socio-cultural background (Meristo and Zeidler, 2022). Specifically, Swedish infants reacted by looking longer at the non-egalitarian distribution, which is consistent with violation of expectations regarding practices of egalitarian distribution that typically characterise large-scale societies driven by abstract, generalised rules. By contrast, Samburu children looked longer at the egalitarian distribution, thereby indicating default expectations of non-egalitarian sharing, which is consistent with a sharing profile based on prior experiences that characterises small-scale societies, as well as the strict hierarchic stratification of Samburu culture. Finally, the Kikuyu children did not differentiate between the two scenarios, a performance pattern reportedly consistent with the Kikuyu culture characterised by less pronounced social hierarchies and increasing WEIRD influences.

Just as reported for joint engagement (Bard et al., 2021), the study of Meristo and Zeidler (2022) shows that socio-cultural influences shape prosocial – and indeed normative – cognition already in infancy. This demonstrates the invalidity of SITh claims of species-wide universality based on the argument that data from infants and toddlers represents a species-universal norm by virtue of representing a stage “before socialisation could have had a major impact” (Warneken and Tomasello, 2009, p. 464). Rather than supporting SITh claims that human altruism is an innate adaptation, the currently available developmental data is consistent with the alternative view that “care and consideration for others is not a given; it develops slowly” (Rochat et al., 2009, p. 417). As already highlighted in Section 3, the cross-cultural data reveals substantial inconsistencies with the developmental outline advanced by SITh, which concern developmental baselines, developmental trajectories, and the developmental timing of traits listed as components of SI. In turn, such developmental inconsistencies entail substantial deviations from the evolutionary narrative advanced by SITh, given that the latter is largely drawn from selective developmental data.

### 4.3. Information sharing and the coordination of cooperation

Communication facilitates the coordination of joint activities, by marking individuals’ motivation (or even their commitment) to engage in specific joint activities, as well as by enabling the synchronisation of joint activity timing. Coordinating joint activities beyond the dyadic level, especially when involving many spatially displaced individuals, as it is the case with group hunting (i.e., the core context in the evolutionary scenario proposed by SITh), would be impossible without communication. According to SITh, “chimpanzees do not actively communicate about the collaboration much or at all” (Tomasello et al., 2012, p. 677), and their vocalisations during group hunting are dismissed as expressions of arousal, with arguments that “chimpanzee vocalisations, as virtually all primate vocalisations, are mostly hardwired to particular stimulus and motivational states, so what is being expressed is general excitement (with the same vocalisation used when excited about other things) and not anything about the content of what is happening or what the vocalizer wants to happen” (Tomasello et al., 2012, p. 677).

In SITh, special attention is paid to commitment as a fundamental motivational force underlying cooperation, and as an expression of a human-unique psychology of obligation (e.g., Tomasello et al., 2012; Tomasello, 2022). According to SITh, children exhibit behavioural markers of commitment in toddlerhood already, while such markers are claimed to be absent in apes. A central piece of evidence for this claim is the finding that in a study where 18-month-olds and 3 zoo-housed juvenile chimpanzees engaged in joint activities with an experimenter who suddenly quit, only the children made attempts to re-engage the reluctant experimenter in the joint activity (Warneken et al., 2006). As argued by SITh, this finding would indicate that young children – but not chimpanzees – understand joint activities as social structures that involve shared goals and a commitment to fulfil shared goals. The children’s attempts to re-engage the reluctant partner are, thus, equated with attempts to re-instate a breached commitment toward a joint goal.

Additional relevant findings are that, in contexts where a joint activity is preceded by commitment, pre-schoolers exhibit more persistent attempts to re-engage a reluctant partner or are more likely to acknowledge their own leaving if forced to interrupt a joint activity (Gräfenhain et al., 2009). In addition, after completing their own goal, pre-schoolers show continued engagement in the joint task until their interaction partners also fulfil their goals (Hamann et al., 2012). Such findings have been interpreted within the SITh framework as evidence that young children appreciate the normative dimensions of collaborative activities and exhibit an understanding of joint commitment and the obligations inherent in social activities with others. Across studies, however, the developmental timing of this ability is not entirely consistent. For example, Gräfenhain et al. (2009) found that 2-year-olds failed to discriminate interactions preceded by commitment from those that were not preceded by commitment, while Hamann et al. (2012) found that 2.5-year-olds did not pursue the joint goal after completing their own goal, thereby showing little concern for their partner’s situation. Moreover, active protests to the experimenter’s interruption of a game were absent in 3- and 4-year-olds (Gräfenhain et al., 2009). These observations suggest that claims about young children’s concern with others’ welfare (see Section 4.2) and about their understanding of commitment (Warneken et al., 2006) may have been overstated. Alternatively, such mixed data may point to unreliable methodology, where small methodological variations may bring about outcomes consistent with substantially different interpretations.

Such methodological and developmental uncertainties notwithstanding, there is robust evidence that, contrary to SITh claims, apes produce a range of communicative signals in a range of joint action contexts. In what follows, we will start by reviewing evidence of communicative signals that facilitate dyadic joint actions (e.g., grooming, social play) including signals that in human children have been interpreted as behavioural markers of commitment. We will conclude the section by reviewing evidence of communicative signals beyond the dyad, used to initiate group travel, to attract other group members to converge toward a specific location, to inform others of dangers, or to facilitate group hunts.

At a dyadic level, apes have been reported to use vocalisations and/or gestures to coordinate the initiation or maintenance of grooming, social play, and mating. For example, chimpanzees coordinate grooming bouts with a specialised multimodal orofacial gesture known as *lip-smacking* (also termed *teeth-clacking*), which is exhibited by the groomer. The gesture consists of a rapid succession of mouth opening and closing, with either the lips or the teeth shaping sound articulation, thus resulting in a smacking or clacking sound. Grooming bouts initiated or accompanied by lip-smacking last longer, thus suggesting that lip-smacking has an affiliative / cooperative function (Fedurek et al., 2015). In a social play context, all ape species exhibit a specialised vocalisation similar to human laughter – the play pant – which has the effect of prolonging play bouts in both chimpanzees and orangutans (Davila-Ross et al., 2008, 2011). Moreover, there is evidence of voluntary motor control over play pant variants, which indicates that such vocalisations may be deployed intentionally and, seemingly, with a cooperative function (Davila-Ross et al., 2011).

Recent data indicate that chimpanzees communicate also for coordinating performance in experimental cooperation tasks. In a recent study by Melis and Tomasello (2019), sanctuary housed chimpanzees were confronted with a collaborative problem-solving task in which a chimpanzee (the communicator) could see the location of tools needed to extract food, while the interaction partner (the recipient) had access to the boxes containing the food, but could not see the location of the tools. In this setup, the chimpanzees were found to quickly develop a successful communication system that allowed them to solve the task.

Recent studies also report data consistent with the presence of behavioural markers of commitment in all ape species. For example, in setups based on the same rationale as the study conducted by Warneken et al. (2006), all ape species have been found to exhibit attempts to re-engage a reluctant partner. Attempts to re-establish coordination have been reported for turn-taking or triadic games with humans and conspecifics, or for intraspecific collaborative interactions such as grooming or social play (chimpanzees, bonobos: Pika and Zuberbühler 2008; MacLean and Hare, 2013; Genty et al., 2020; Voinov et al., 2020; Heesen et al., 2021; gorillas: Tanner and Byrne, 2010; orangutans: Gruber, 2013). Recall that, according to SITh, engaging in mutual gaze and / or gestures when joint action is perturbated indicates an understanding of collaborative roles, shared motivations, and joint commitment. Interestingly, in ape species, the contextual variability of such communicative behaviours bears some resemblance to that reported in humans. First, activity resumption is primarily initiated by the individual responsible for activity interruption. Second, overt signalling is modulated by social distance and power differences, being more frequent in interactions between less bonded or differently ranked individuals compared to strongly bonded individuals or same-rank individuals. Third, such signalling behaviours occur before the initiation and termination of collaborative activities (Genty et al., 2020; Heesen et al., 2021).

Evidence of joint action coordination through communication beyond the dyadic level has been reported in several contexts, encompassing alerting of dangers, foraging, group travel, group resting, and group hunting. As already mentioned, chimpanzees and bonobos inform ignorant conspecifics about potential dangers with acoustically distinct alert *hoos* – a behaviour which is currently used as a measure of intentional cooperation (Girard-Buttoz et al., 2020). Another context-specific vocalisation linked to joint action beyond the dyadic level is the so-called ‘travel *hoo’,* which chimpanzees produce prior to departure, seemingly to recruit travel partners (Gruber and Zuberbühler, 2013). The travel *hoo* is reported to result in high rates of recruitment success, which supersede recruitment rates with silent means (e.g., tactile gestures). Moreover, the travel *hoo* satisfies the criteria for intentional communication, as it is directed at a target (typically allies), accompanied by monitoring behaviours (so-called ‘audience checking’) and produced persistently if the audience does not respond. In a resting context, chimpanzees produce so-called ‘rest *hoos’* – acoustically distinct vocalisations whose effect is the prolongation of resting time (Bouchard and Zuberbühler, 2022a). Rest *hoos* may be answered in kind by conspecifics, and such dialogic (and polylogic) bouts of rest *hoos* have been reported to increase even further the duration of resting. Similar to the travel *hoos*, rest *hoos* also meet the criteria of intentional communication.

In a plant-food foraging context, chimpanzees typically produce two types of calls: context-specific, close-range ‘food (rough) grunts’ and long-range ‘pant hoots’. The ‘pant hoots’ produced in this context appear to function as a cohesion call, as they are targeted at distant allies, informing them of the presence of food (Bouchard and Zuberbühler, 2022b). Food grunts have been proposed to have an informative (referential) function, as acoustically different grunts have been found to be associated with different food qualities (Slocombe and Zuberbühler, 2006; Watson et al., 2015). Food grunts may be selectively directed at valuable social partners (Slocombe et al., 2010), a context where they appear to function as a signal for initiating or prolonging feeding with selected social partners (Fedurek and Slocombe, 2013). A recent study, however, found that food grunts produced by low-rank males in the presence of dominants may have an appeasement (and thus competitive) function (Bouchard and Zuberbühler, 2022b).

Finally, in the context of group hunting, chimpanzees produce acoustically distinct and context-specific barks (Crockford and Boesch, 2003). These barks occur prevalently prior to the initiation of hunts and, based on recent data, they are associated with more efficient cooperative efforts (Mine et al., 2022). Hunting barks may function to indicate the individuals’ motivation to engage in hunting, as individuals who barked prior to hunt initiation were more likely to pursue prey. Hunting barks appear to also have a recruiting function contributing to motivating more individuals to join the hunt. Finally, barks may also help individuals to coordinate more efficiently, thereby resulting in reduced hunt duration.

Summing up, the data reviewed in this section show that, contrary to SITh claims, non-human apes exhibit a considerable repertoire of gestures and vocalisations deployed prior or during joint activities. Statistical analyses demonstrate that such behavioural signals have a coordination function, typically regulating activity initiation, continuation or interruption. The functional contexts in which these signals are used vary from grooming and play coordination in dyads, to the coordination of group travel, resting, plant foraging and hunting. In addition, in novel contexts that require action coordination such as it is the case of certain experimental setups, apes can settle on a communication system that allows them to solve a cooperation problem. For nearly all the signals reviewed in this section, behavioural and statistical analyses confirm that these signals fulfil criteria of intentionality. Finally, a subset of these signals has been recorded in contexts where they would fulfil SITh criteria for behavioural markers of commitment. Signals used in such contexts function to re-instate an interrupted joint activity or announce an interruption, and exhibit forms (e.g., mutual gaze, gestures) and contextual features reminiscent of those described in humans.

In a recent reiteration of SITh claims concerning action coordination through communication and commitment (Tomasello, 2022), such ape signals have been dismissed as merely indicating a preference for engaging in a rewarding activity or, more broadly, as a preference for engaging in a social – as opposed to solitary – activity. In this rebuttal, the list of acceptable markers of commitment is shifted toward criteria such as continuing a joint activity until the partner also reaches their goal, protesting when the partner interrupts a joint activity or apologising when interrupting an activity. Note, however, that the updated criteria rely heavily on verbal communication, which are of little use in a comparative context. Moreover, with these criteria in place, the claims of SITh concerning the status of commitment as a biological adaptation would require some revision. As we reviewed in the beginning of 4.3, the performance of children with a WEIRD socio-ecological background begins to be consistent with such criteria starting in the preschool years. Moreover, it remains unknown if the WEIRD data constitute the norm for the human species, as it remains unknown whether – and if so, when – children with other socio-ecological backgrounds fulfil such criteria. A limitation of currently available data on communicative signals of action coordination is that most of the human data comes from WEIRD samples and most of the ape data comes from studies with chimpanzees, and to a less extent with bonobos. Our understanding of the evolution of human cooperation will benefit from more research conducted with bonobos, gorilla and orangutan species, as well as with non-WEIRD samples.

## 5. The imitation and cumulative culture cluster: the social side of imitation

The significance of imitation within SITh is primarily related to the phase of *collective intentionality,* when a need for ingroup identification is hypothesised to have emerged in a context of increased group-size and fierce inter-group competition. It is further argued that this need was initially solved through the conventionalisation of behavioural practices, whereby group-specific ways of doing things (e.g., hunting, food processing, communicating) served as group identity markers (Tomasello et al., 2012; Tomasello, 2014, 2018a). Since behaving according to group conventions is postulated as mandatory for survival, this arguably created adaptive pressures for the emergence of socio-cognitive mechanisms that facilitated the transmission of group-specific ways of doing things. Such mechanisms include social learning through imitation, conformity to group norms, and teaching, thereby leading up to cumulative cultural evolution, as well as the emergence of institutions that ensure the preservation of group-level culture by enforcing group-level conformity to group norms (Tomasello, 2018a, p. 666).

Arguments pertaining to the imitation and cumulative culture cluster invoke data suggesting that (i) in imitation recognition studies, apes do not exhibit signs of playfulness and enjoyment (as children do), and in imitation learning experiments apes (ii) gaze less than human children at the experimenter’s face; (iii) they emulate rather than imitate, i.e., they primarily copy demonstrated goals but not the demonstrated actions to achieve those goals; (iv) they do not ‘overimitate’, i.e., they do not copy perceivably irrelevant actions to achieving the goal, and as a consequence apes (v) may exhibit some cultural variation in the form of behavioural practices, but they do not exhibit cultural practices, where the difference between behavioural and cultural practices is that the latter are *explicitly* shared at group level as conventions, based on a (more or less explicit) *agreement* to do things a certain way and on collective expectations that everyone else in the group ought to behave the same way (Tomasello et al., 2012). Note, however, that this argument builds primarily on the SITh claim that apes are not capable of recursive mind reading, which according to SITh, is the human-unique trait that affords an objective perspective and, thus, normative cognition (Tomasello et al., 2012). In what follows, we will examine each of the five features listed above, which sometimes are referred to collectively as ‘the social side of imitation’ (for reviews and discussions see Carpenter and Call, 2009; Nielsen, 2009; Tomasello et al., 2012).

In imitation recognition studies, participants interact with an experimenter who imitates all their actions. In control conditions, participants are variably exposed to periods of no action, pre-established action sequences that are non-contingent in relation to the participants’ actions, non-imitative actions that are contingent upon the participants’ actions, or imitative actions with suppressed emotional responding (for an overview, see Sauciuc et al., 2020). Behavioural responses – typically visual attention to the experimenter, positive affect, testing behaviours and, sometimes, tendencies to increase proximity to the experimenter – are then measured and compared across conditions. Visual attention, positive affect and smiling are considered implicit measures of imitation recognition, that indicate whether participants unconsciously discriminate the imitation condition from the other conditions. In contrast, testing behaviours are considered an explicit measure of imitation recognition, whereby participants that produce testing behaviours (persistent repetition of an action or rapid sequences of different actions while monitoring the experimenter) exhibit a conscious awareness of being imitated. Testing behaviours may give rise to so-called ‘imitation games,’ whereby actions are repeated or imitated back-and-forth between the participant and experimenter.

Behavioural markers of enjoyment (smiling, laughter) and playfulness (imitation games) in response to being imitated have been proposed as markers of shared intentionality, and their reported absence in apes has been interpreted as an indication that apes do not understand – and do not share – the cooperative intentions of the interaction partner (Nielsen, 2009). This argument, however, is based on a single-case study (Nielsen, 2009), which is currently invalidated by alternative evidence (Persson et al., 2018). The naturalistic observations collected by Persson et al. (2018) show that zoo housed chimpanzees may spontaneously engage in simple imitation games with the visitors that could last for up to 19 turns. In this context, only familiar actions have been observed to be copied (by both apes and humans), thereby precluding a learning function for behaviour copying, and this form of social imitation had the effect of prolonging cross-species interaction. Occasionally, the chimpanzees also exhibited play face or laughter when their behaviour was imitated by zoo visitors (Persson et al., 2018). In addition to the positive findings above, it is worth mentioning that several examples reported in social-game interruption studies (reviewed in 4.3.) were arguably also imitative in nature, such as throwing an object back-and-forth. We note that a recent attempt to replicate the findings of Persson et al. (2018) found only negative results (Motes-Rodrigo et al., 2021). Two crucial methodological differences between the original and the replication study may explain their contrasting results. First, unlike the naturalistic approach of the original study, the replication used informed human participants who were aware of being video recorded. Second, the replication study targeted imitation learning of bodily actions, while the original study focused on social imitation, i.e., the imitation of familiar, mundane actions. With this adjusted method the replication not only failed to find imitation (by either species), but also hardly recorded any chimpanzee action directed toward humans, compared to over 1,500 occurrences in the original study. Crucially, as reported in Persson et al. (2018), the prevalence of both cross-species interaction and imitation varied both among chimpanzee individuals and visitor categories. This highlights that, in both species, motivation was crucial for engaging in social interaction in general, and social (non-learning) imitation in particular. The relaxed, voluntary, and often playful interactions that we believe foster social responses in chimpanzees toward strangers was the reason we did not recruit and filmed informed zoo visitors, but instead used a classic, pen-and-paper naturalistic observation approach.

The second pro-SITh argument related to the social side of imitation, whereby children are reported to look longer than apes at the experimenter’s face in imitation learning tasks, would indicate that apes lack the motivation to grasp (and thereby share) the states of interaction partners (Carpenter and Call, 2009). First, note that this argument highlights a quantitative and not a qualitative difference, suggesting that the apes are not entirely unmotivated to enter visual joint engagement in such tasks. Second, before drawing conclusions about the socio-cognitive implications of looking time differences between humans and apes, known visual scanning differences between the tested species need to also be considered. Indeed, (zoo-housed) apes shift their fixations more broadly and quicker than (WEIRD) adult humans (for a recent review, see Lewis and Krupenye, 2022), who, in turn, have significantly quicker fixations than (WEIRD) children aged 2–6 years (Helo et al., 2014). These between-species differences with respect to scanning and duration of fixation have been linked to search requirements imposed by species-specific environments. As such, faster and broader scanning enables apes to quickly process many stimuli, which is advantageous in their typical habitat to handle unpredictable encounters with predators or unfamiliar conspecifics. The longer fixations of children, on the other hand, have been linked to cognitive underdevelopment, whereby children require more time to process visual information (Helo et al., 2014 and references therein). Species-specific biases may also explain differences in looking time, since, except for bonobos, all ape species attend longer to conspecific as opposed to human faces. Furthermore, apes may avoid longer fixations on faces, since this may be perceived as a threatening or sexual signal, etc. (Kaplan and Rogers, 2002). Given all these alternative explanations, looking time differences at the experimenter’s face is not an unequivocal argument for cross-specific socio-cognitive differences.

Against the third argument that apes only emulate (i.e., copy goals) and never ‘truly’ imitate (i.e., copy the form of bodily actions) there is evidence that apes can imitate the form of bodily actions, at least in some contexts. For example, in studies using the ‘Do-as-I-Do’ paradigm, several chimpanzees and one orangutan were first trained to copy a variety of human actions on command and subsequently tested with a variety of novel actions (Custance et al., 1995; Call, 2001; Pope et al., 2018). The results showed a considerable level of action matching by *both enculturated and non-enculturated apes*. Moreover, the more detailed data analysis conducted in one of the studies also showed that the majority of imitative responses were cases of full (as opposed to partial) imitation (Call, 2001). Interestingly, partial imitation was prevalent for high-detail actions (e.g., finger gestures), leading to the suggestion that apes may have a difficulty discriminating fine grained actions.

Other examples come from the ‘rational imitation’ paradigm where participants are presented with a range of means for creating the same results (e.g., turn on a light with the head, foot or bottom) in two conditions: experimenter has free hands vs. experimenter has occupied hands. In this setup, participants exhibiting ‘rational imitation’ are expected to imitate only in the condition when the experimenter’s hands are free, which suggests that the experimenter is demonstrating ways to do things, while in the ‘hands tied’ condition there is an alternative explanation for the strange behaviour of the experimenter. In line with these predictions and similarly to human children, chimpanzees were found to use the same way of turning on the light as demonstrated by the experimenter, but only when the experimenter’s hands were free (Buttelmann et al., 2017).

Examples of imitation have also been reported in (semi-)wild populations. The studies by Russon and Galdikas (1993, 1995) are now classical examples of novel action (i.e., beyond species-specific behavioural repertoire) imitation in free-ranging rehabilitant orangutans, which attempted to copy a range of human behaviours. Another compelling example is the case reported by Hobaiter and Byrne (2010) where the unusual scratching technique of a snare-injured chimpanzee with almost full hand disability was copied by fully able individuals. Since the behaviour was selectively copied by individuals who associated frequently with the injured chimpanzee, the spread of the novel scratching technique can be explained by social learning: a case of bodily action imitation. These examples are valuable given criticisms that all extant evidence of body form copying by apes lacks ecologically validity due to enculturation effects or prior training, or represents copying of actions that the apes could produce even without exposure to a model (see, e.g., Neadle et al., 2021 and references therein).

While the findings summarised above challenge SITh claims concerning the absence of (bodily form) imitation in apes, other findings challenge claims concerning the putative selection pressures promoting the evolution of imitation, and the adaptive significance of learning through (bodily action) imitation. Recall that, according to SITh, social learning through faithful (action) imitation – as opposed to social learning through emulation – is a cooperative process enabled by SI, whose adaptive significance resides in enabling the faithful transmission of group-specific ways of doing things, ultimately leading to cumulative culture evolution. However, contrary to this contention, chain transmission experiments indicate that emulation is a highly efficient mechanism of technological transmission, although imitation may provide some advantages for the transmission of relatively complex and causally opaque designs. For example, both human adults and children successfully pick up specific features and styles of technological designs (e.g., paper airplanes, spaghetti towers) by simply witnessing final products of predecessor groups, which then get passed on – and may also cumulatively improve – to successive chains of participants without any use of imitation (reviewed by Heyes, 2021, 2023). While imitation has been found to be required for acquiring some skills, its contribution to the inheritance of highly complex skills, such as stone tool making, appears to be rather insignificant (as reviewed by Stout and Hecht, 2017). Studies on the latter suggest that knapping techniques pose significant demands on the calibration of perceptual, attentional and motor abilities concerning striking efficiency rather than on copying the right body form (idem). Such protracted learning through calibration of embodied skills is evident in the acquisition of basic ‘flaking’ that corresponds to Oldowan tool knapping (dated to *H. habilis* some 2.6–1.5 mya), but even more so in the acquisition of more advanced techniques, such as hand-axe knapping (dated to *H. erectus* and *H. heidelbergensis*, between 1.7–0.25 mya) or ‘prepared core’ flaking (dated to *H. neanderthalensis and H. sapiens*, some 0.25 mya).

The evidence reviewed above suggests that the contribution of imitation to the inheritance of instrumental actions in humans is rather limited: on the one hand, emulation is an efficient inheritance mechanism for (relatively) simple technological designs, on the other – imitation is inefficient for the inheritance of complex technology. Given such findings, an alternative proposal is that imitation – where focus primarily lies on action form – may be indispensable for the transmission on non-instrumental behaviours, i.e., communicative and ritualistic behaviours (Heyes, 2021, 2023). In this view, the capacity for imitation is based on relatively simple cognitive mechanisms that enable the formation of self-other correlated sensorimotor associations through experience, and is promoted by complex cultural environments that provide a rich supply of such experiences. Such an alternative view bypasses both the conceptual and empirical challenges to SITh discussed above, and has the potential of reconciling evidence of (action form) imitation and behavioural traditions in apes – as well as other nonhuman species (for a recent review, see Zentall, 2022) – with claims of human uniqueness concerning social learning and cumulative culture evolution.

The fourth claim that apes do not exhibit overimitation is likely to be the most compelling claim of SITh, as evidence is currently lacking that apes copy actions that are perceivably irrelevant to achieving a demonstrated goal. In tasks typically used in overimitation experiments, chimpanzees appear to take a more rational stance compared to children. Specifically, if actions and goals are demonstrated on an apparatus where the causal relationship between actions (manipulations on the apparatus) and goals (obtaining the reward inside the apparatus) cannot be perceptually determined, chimpanzees copy all demonstrated actions at levels comparable to those of children (Horner and Whiten, 2005). However, if the demonstration is done on an apparatus which reveals that some of the demonstrated actions are not necessary for attaining the goal, apes tend to selectively imitate the efficient actions, while children continue to imitate both relevant and irrelevant actions. As a side note, it is important to highlight that, traditionally, overimitation studies focus on whether participants copy goal-relevant or goal-irrelevant actions, while paying little attention to whether they do so through emulation or (action form) imitation. According to SITh, the performance pattern described above indicates that apes imitate actions only when driven by selfish goals, i.e., when the imitated actions are necessary for retrieving a food reward. In turn, this would suggest that they have the cognitive capability for faithful imitation, but they lack the social motivations to do so. Conversely, the propensity of children to imitate both goal-relevant and goal-irrelevant actions would suggest that children do so for social reasons, such as showing that they share something with others, which, in turn would serve affiliation and identification purposes (Carpenter and Call, 2009; Over and Carpenter, 2013).

Cross-cultural evidence suggests, however, that this interpretation should be re-evaluated, as children’s propensity for slavish imitation may not be a human universal. For example, empirical data show that overimitation is nearly absent among Aka children of the Congo Basin, although it is present among adults of the same hunter-gatherer culture (Berl and Hewlett, 2015; Hewlett et al., 2016). This undermines the contention that overimitation is a human-specific biological *adaptation* that enabled the evolution of cumulative culture, and is consistent with alternative views on overimitation – and, more broadly – high-fidelity imitation. Accordingly, overimitation may reflect a practical ‘copy-all, refine later’ rule of thumb for acquiring useful techniques in an environment populated with causally opaque artefacts and practices (Whiten et al., 2005a). Accordingly, populations that exhibit a causally transparent cultural-environmental niche (chimpanzees, Aka children) tend to emulate in the *transparent* condition of the *opaque-transparent paradigm* mentioned above. Indeed, as Berl and Hewlett (2015) noted, the functions of the artefacts that young Aka children use or observe being used daily are easy to recognize. Likewise, obvious tool functionality predominates in the material culture of the chimpanzees, although not exclusively characterising it (Whiten, 2019, 2022; Boesch et al., 2020). Conversely, the presence of overimitation among Aka adults is consistent with an increased opacity of both cultural and instrumental activities from adolescence onwards, when subadults begin to acquire complex skills such as basket weaving or spear hunting, as well as complex social practices, such as participating in initiation ceremonies (Berl and Hewlett, 2015; Hewlett et al., 2016).

The study of Berl and Hewlett (2015) provides yet another interesting angle on the ultimate functions and proximate mechanisms of overimitation, given that childhood over-imitation is reported for the Ngandu – an agrarian population residing in the vicinity of Aka territories. The differences between the Aka and the Ngandu children can, thus, also be explained by socio-ecological differences. Aka children are socialised in a hunter-gatherer culture that emphasises autonomy and egalitarianism, being thereby characterised by minimal deference for the elders and by low normativity (Berl and Hewlett, 2015; Hewlett et al., 2016). As Berl and Hewlett (2015) note, the Aka rarely tell others, including children, what to do. In contrast, the Ngandu enforce age and gender social hierarchies, as well as social relationships based on economic or material components, as opposed to the purely emotional bonds that forge Aka relationships. This may suggest that children’s overimitation is promoted in cultures that enforce social hierarchies (including deference for the adults) and (normative) conformity. Indeed, consistent with this proposal, Clegg et al. (2017) found higher levels of overimitation among Ni-Vanuatu children compared to American children, a result which is argued to reflect the higher valuation of conforming behaviour among Ni-Vanuatu parents compared to American ones.

The proposal that the expression of overimitation is positively influenced by conformity and adult deference is further supported by a range of contextual effects from experiments with WEIRD samples. These experiments indicate that children may exhibit heightened levels of overimitation if the experimental situation can be interpreted as a situation that requires compliance with implicit social demands, such as a pedagogical or as a play situation (for recent reviews of overimitation studies and contextual effects, see Hoehl et al., 2019; Whiten, 2019). As such, the more obviously causally irrelevant actions are, the more likely children will interpret them as behavioural norms or game rules (Marsh et al., 2014), with the consequence that overimitation may vanish completely if the experimenter declares the experiment concluded (McGuigan and Robertson, 2015). In the same vein, overimitation levels co-vary with perceived social pressure, as children show low levels of overimitation if the experimenter leaves the room after demonstrating the actions, and are less likely to overimitate if the demonstrator is a low-authority individual, such as a same-aged or younger peer (McGuigan et al., 2011; Wood et al., 2012, 2013; Zmyj and Seehagen, 2013). However, they will overimitate peers if the experimental situation is framed as play (Wood et al., 2016). Taken together, this body of data has led to the proposal that overimitation is a behaviour triggered by the perception of demonstrated actions as prescriptive norms (Kenward et al., 2011). It must be noted, however, that even WEIRD samples may fail to exhibit overimitation and the normative stance hypothesised to underlie overimitation. For example, several studies have now documented a preference for efficiency over normativity in children aged 2 to 6 years, whereby children choose achieving a goal with a more efficient tool rather than a normative tool, the latter being operationalised as the tool that has been used by several models or that has been described as being preferred by a majority (e.g., Fong et al., 2021 and references therein).

Overall, the developmental findings reviewed above suggest that overimitation is a phenomenon driven by a range of contextual factors related to technological opacity and conformity rather than (simply and solely) a motivation to share states. While conformist tendencies are consistent with a desire to affiliate, it is currently not possible to determine if this is driven by individualistic or altruistic motivations. In fact, overimitation can be conceived of as a special case of the more encompassing category of conformity, where (e.g., behavioural) preferences of the social group are endorsed by an individual, such as in so-called conformist transmission (Henrich and Boyd, 1998) and may even override pre-existing personal preferences, such as in so-called Aschian conformity (Asch, 1955, 1956). Since the core feature of conformity is copying the way one’s social group does things, we previously proposed the addition of conformity to the class of behaviours pertaining to the ‘social side of imitation’ (Persson et al., 2018). In the same vein, Whiten (2019) pointed out important similarities between the two constructs (overimitation and conformity), in that both share ‘a disposition to yield or to comply with others’ (p. 33). However, a key feature that differentiates conformity from overimitation is the number of models: whereas conformity is established after observing a group majority or, at least several conspecifics, overimitation is elicited after observing a single model. Interestingly, the presence of conformity phenomena has been attested in a variety of taxa, ranging from insects to non-human primates (as reviewed by Whiten, 2019). Importantly, non-human primates evidence both conformity transmission, as well as Aschian conformity where personal preferences are overridden by social preferences (for examples, see below), thereby suggesting that apes too can copy behaviours for social, potentially affiliative reasons.

Related to the above point, and arguing against the fifth SITh claim, evidence has now accumulated that, in all ape species, there is considerable within-species variability and group-level specificity with respect to foraging techniques, social practices and communicative signals. This body of research strongly suggest the implication of high-fidelity social learning in the between-individual and cross-generational transmission of a multitude of practices. For example, neighbouring bonobo communities may exhibit diverging food preferences, despite similar ecology, frequent inter-group encounters and occasional between-group food sharing (Samuni et al., 2020). Chimpanzees exhibit a wide range of group-specific termite-fishing techniques, which vary across populations with respect to the type of tool, movement, or action sequencing – a variation that, again, cannot be explained by ecological constraints (Boesch et al., 2020). In addition, they exhibit highly stable group-specific variants of arbitrary social conventions, as exemplified by group-specific patterns of grooming or greeting customs (Nakamura and Uehara, 2004; van Leeuwen, 2021; Girard-Buttoz et al., 2022). Moreover, group-specific ‘dialects’ have been documented by several studies, with between-group variability being identified at the ‘phonetic’ level of acoustic features (e.g., frequency, inter-call interval, Watson et al., 2015) or at the ‘phonotactic’ level of element sequencing (Girard-Buttoz et al., 2022). Considerable variability and group-specificity of behavioural practices (van Schaik et al., 2003) and vocal ‘dialects’ (Wich et al., 2012) has been also documented in orangutans.

Such within-population homogeneity and cross-population diversity of practices is difficult to explain in the absence of high-fidelity social learning. While group composition changes constantly, through the affluence of immigrant individuals, group-level practices exhibit considerable stability, even when the ecological constrains are identical across groups. This indicates that immigrant individuals swiftly abandon practices acquired in their group of origin and adopt the practices of their destination group (for recent reviews see Whiten and van de Waal, 2018; Whiten, 2022). This includes also vocal learning as a result of migration (Watson et al., 2015). The presence of learned traditions is also confirmed by experimental studies, where alternative forms of actions are seeded across experimental groups of (primarily) chimpanzees. Such arbitrarily seeded ‘ways of doing things’ tend to spread with remarkable fidelity and are maintained even when competition with alternative ‘ways of doing things’ is introduced (Whiten et al., 2005b; for more examples and recent reviews, see Whiten and van de Waal, 2018; Whiten, 2022).

Overall, observational and experimental data reveal apes’ potential for considerable conformity and suggest that social motivations theorised to drive conformity and overimitation in humans may be shared with apes. It moreover suggests that a key aspect emphasized by theories of the evolution of cumulative culture is present in apes: group-level stability of practices through high-fidelity social learning and conformity. This may, in fact, be present at a level that blocks the transmission of more efficient innovations, which is the other key aspect of cumulative culture evolution. While chimpanzees recognise tool or technique efficiency, can easily acquire novel foraging techniques as shown by seeding experiments, and are capable of innovating (for reviews see Whiten and van de Waal, 2018; Whiten, 2019, 2022), the spreading of innovations is blocked by conformist tendencies linked to pre-existing practices, but also by rank-based transmission biases. Among chimpanzees, the main sources of innovation, i.e., juveniles and immigrants, are typically low-ranking individuals, which are less likely to be copied by others.

Evidence is currently accumulating that ape traditions are maintained by additional transmission mechanisms besides conformity and copying dominants, suggesting a significant degree of overlap with those found in humans (as reviewed by Whiten and van de Waal, 2018). As such, both humans and apes exhibit an initial bias of several years, where the acquisition of practices is dominated by maternal models (for recent reviews see Maestripieri, 2018; Whiten and van de Waal, 2018). In a subsequent phase, human and ape immatures switch to a preference for other within-group models, with a bias to learn from those perceived to have more success or skills (humans: Henrich and Broesch, 2011; chimpanzees: Vale et al., 2014; Kendal et al., 2015). Immatures may also exhibit age-related or sex-related biases, with a preference to learn from older models (humans: Henrich and Broesch, 2011; chimpanzees: Biro et al., 2003) or from same-sex models (humans: Henrich and Broesch, 2011; orangutans: Ehmann et al., 2021).

The transmission mechanisms reviewed thus far focus on learners’ motivation to acquire group-specific practices as an expression of the ‘social side of imitation’. The perspective of the models (or demonstrators), however, has been less extensively studied in apes, and it is currently unclear if ape models intentionally and actively contribute to the transmission of cultural practices. Based on SITh, ape models are passive, while humans have evolved active modes of imparting knowledge and eliciting social learning in learners, such as ostensive communication and teaching, which are based on shared intentionality.

In humans, information sharing is often preceded by so-called ostensive signals, e.g., pointing, looking, vocalising. Ostensive communication is hypothesised to facilitate social learning, by activating expectations that the subsequent information will be of relevance to the recipient, and thus worth acquiring – a phenomenon dubbed ‘natural pedagogy’ (Gergely and Csibra, 2013). Indeed, from 18-months of age, human infants are more likely to imitate after being exposed to ostensive cues (Southgate et al., 2009) and prefer problem-solving methods preceded by ostensive cues even when such solutions have been demonstrated to be less efficient (e.g., Király et al., 2013; Marno and Csibra, 2015). Interestingly, a similar bias is found in chimpanzees, bonobos and orangutans, who also endorse the less efficient solution when the demonstration is preceded by ostensive cues (Marno et al., 2022). Notably, in the study of Marno et al. (2022), choosing the less efficient solution entailed a 50% reward loss compared to other conditions where an efficient tool was selected. This performance pattern suggests that apes follow ostensive signals even when these go against their own experience and entail foregoing a sizeable proportion of resources.

The propensity of apes to tailor what to learn based on ostensive communication may seem puzzling against the background that model influences on practice acquisition are exerted passively. That is, the contribution of the adult models is merely to tolerate the proximity of immatures, who then acquire foraging and social practices by observing the models. The primatological literature, however, contains several examples in which ape mothers appear to take a more active role in the immatures’ learning, through interventions reminiscent of behaviours that in the human literature are categorised as scaffolding. Such behaviours are found to channel skill acquisition by facilitating or hindering exploration. In humans, scaffolding is contingent upon the so-called zone of proximal development, indicated by the fact that the interventions of the expert depend on the knowledge level of the learner, whereby the ‘scaffold’ is gradually removed, as the learner becomes more proficient (e.g., Palincsar, 1986).

In the nonhuman literature, scaffolding behaviours have been described as a form of functional teaching, whereby experts behave in a way that facilitates learning in novices, but their behaviour is not driven by explicit intentions to cause an individual to learn (e.g., Caro and Hauser, 1992). Several forms of scaffolding have been reported in apes, including maternal encouragement, discouragement, parallel action engagement, co-action, and tool transfer (e.g., Nishida, 1987; Boesch, 1991; Moore, 2013; Musgrave et al., 2019; Sauciuc et al., 2021). Some of these strategies may apply flexibly across a range of contexts and skills to be acquired, which contrasts with reports from other taxa, and is reminiscent of the flexibility and generalisability that characterise human functional teaching, which, at least in some contexts, is intentional (e.g., Caro and Hauser, 1992; Moore, 2013; Lew-Levy et al., 2017; Boyette and Hewlett, 2018). That apes may exhibit intentional scaffolding is currently an intriguing possibility, given evidence of (arguably) intentional information sharing in other contexts (as reviewed in Sections 3.1 and 4.3). Note, however, that explicit intentionality is not warranted in human functional teaching, and that formal teaching in the shape of explicit instruction does not appear to be a human universal. In fact, even functional teaching may be rare in some hunter-gatherer communities, as the prevalent learning strategies in such populations are those that favour children’s autonomy to explore and experiment with minimal interference from the adults (for recent reviews see Lew-Levy et al., 2017; Boyette and Hewlett, 2018). Interestingly, in said hunter-gatherer communities, a domain more likely to implicate structured – and plausibly intentional – forms of instruction during childhood is the acquisition of norms regarding sharing resources. Explicit forms of teaching are otherwise reported to occur primarily for the acquisition of complex (and opaque) skills and rituals during adolescence, although even at these ages the primary modes of acquisition remain observation, trial-and-error practice, and participation.

Summing up, the comparative and developmental evidence reviewed in this section dedicated to the ‘social side of imitation’ cluster reveals once more the pattern noted above for the other two clusters. On the one hand, there is positive evidence on the presence of putative SI components in nonhuman apes, as judged by criteria formulated in SITh. On the other hand, the developmental data fail to validate the developmental claims of SITh. Traits and / or performance aspects that SITh presents as species-specific and species-universal in human ontogeny are instead characteristic of a behavioural phenotype, as they fail to materialise cross-culturally. Before concluding this section, it is important to note that SITh argumentation concerning the social side of imitation relies primarily on data obtained in social learning contexts, thereby overlooking categories of behaviour-matching in purely social (i.e., non-instrumental) contexts. This contrasts with perspectives from developmental and social psychology, where the domain of social imitation extends to non-instrumental imitative phenomena such as non-conscious mimicry, overt communicative imitation, mimicry- and imitation-induced prosociality, and behavioural synchronisation. All these additional aspects of the ‘social side of imitation’ have been documented in apes, other nonhuman primates or even non-primate species (as reviewed in Persson et al., 2018; Whiten, 2019). Addressing the full range of imitative phenomena known to exert social functions will be necessary in future research in order to draw a more complete picture of the types, extent, functional and proximate mechanisms of joint activities and joint engagements, i.e., of behavioural phenomena that lie at the core of SI.

## 6. Conclusion

Cooperation defines humanity – for good and for bad. It allows us to enjoy collective pastimes (from children’s games to sports and music), it enables global charity networks, but it can also deflect humans into advanced warfare. Understanding the evolution of human cooperation, its functional and mechanistic underpinnings, is thus essential for understanding the nature of human social behaviour and the societies we form. In the present review we examined the claims of a highly influential theory on the evolution of human cooperation – the Shared Intentionality Theory (SITh) – with the purpose of giving visibility to a body of data that is inconsistent with it. SITh proposes that human cooperation is enabled by a suite of species-specific socio-cognitive abilities and motivations – collectively termed shared intentionality (SI) – that enable the mutually aware pursuit of shared goals during joint activities. SITh builds around the core tenet that, due to a putative shift to obligate interdependent foraging set to have happened about 400,000 years ago in *Homo heidelbergensis*, humans have evolved a unique kind of altruistic psychology that is based on SI and specifically adapted for cooperation. In contrast, the social behaviours of our closest living relatives – the nonhuman apes – are said to exclusively be driven by an individualistic psychology encompassing skills and motivations evolved for competition.

SITh claims are defended with empirical data from a series of socio-cognitive experiments carried out with apes and / or human children of various ages, and performance differences between the specific studied groups are presented as species-level differences. The developmental outline derived from this set of studies, encompassing claims about the onset, trajectory, and contextual features of SI-relevant skills and motivations, is central for deriving the evolutionary scenario advanced by SITh. The developmental outline proposed in SITh influences, in particular, the theoretical scaffolding surrounding the evolutionary emergence and co-dependency of abilities attributed to the SI-complex, with abilities argued to emerge early in infancy being hypothesised to have emerged early in the evolution of SI.

As we repeatedly noted in our review, even in the studies selected to argue in favour of SITh, the results are sometimes mixed and performance differences are often quantitative rather than clear-cut qualitative. These observations would, in fact, suffice to mitigate the radical claims of SITh and contend that the radical divide between ape and human psychology (proposed by SITh) may, in fact, be a continuum. And regardless if “continuum” is a good or bad descriptive metaphor, future research does not benefit from simplified species characterizations. This alternative view finds additional – and robust – support in the plethora of SITh-inconsistent findings reviewed in our paper. The comparative data that contradicts SITh claims can rightfully be described as systematic, rather than limited to exceptional cases, as it spans the main thematic clusters of SITh: recursive mind reading (posited as a precondition for SI), cooperation and altruistic behaviour, and the so-called social side of imitation. Overall, the findings reviewed above are inconsistent with SITh claims that ape social cognition exclusively encompasses abilities evolved to successfully compete with others (e.g., Tomasello et al., 2012; Tomasello, 2018a,b). To the contrary, these findings show that ape social interactions are not blindly driven by selfish goals and competition, nor are apes blind to cooperative intentions. The empirical data demonstrate that apes can engage in cooperation, make costly prosocial choices (especially toward others who provided them with benefits), grasp the cooperative intentions of others, have some form of an understanding of commitment, and exhibit an intolerance of freeloaders. Such findings expose normative dimensions in the social conduct of apes. Indeed, traits such as prosocial concern, inequity aversion, and third-party punishing interventions have been recently discussed as evolutionary building blocks of human moral norms that may be present in nonhuman animals (Burkart et al., 2018). Taken together, this body of research suggests that traits associated with the SI-complex and altruistic psychology have ancestral origins, some of them going (at least) as far back as to the emergence of the *Hominidae*. While we certainly do not contend that human social cognition exhibits species-unique features, several of the features proposed in SITh, specifically, fail to fulfil the human-unique criterion given evidence consistent with their presence in nonhuman apes.

In our review we also repeatedly highlight that several developmental traits presented as species-characteristic and as distinguishing human from ape psychology do not represent the norm for *Homo sapiens* as a species. This is again a systematic occurrence rather than being a limited collection of exceptional cases. Paralleling the comparative data, the developmental data exhibits cross-cultural variability for all the thematic clusters associated with SI. When judged through the lens of the behavioural criteria and developmental outline proposed by SITh, this variability would place (sometimes a large majority of) human cross-cultural samples outside the human psychological norm. The developmental picture drawn in SITh is – to a large extent – representative of the WEIRD phenotype, but frequently does not appear to extend beyond this phenotype. Thus, since the set of criteria proposed in SITh reflects the WEIRD phenotype but is not representative of *Homo sapiens* as a species, claims of species-level differences derived from comparisons between the WEIRD phenotype and specific ape populations have dire validity problems. Given the presence of cross-cultural data that invalidates the developmental outline advanced in SITh – including the onset, trajectory, sequence, and dependencies of abilities in the SI-complex – then, by necessity, the evolutionary scaffolding which rests on this developmental outline is also undermined.

Overall, this is symptomatic of a pervasive limitation in comparative research, where species-level conclusions tend to be drawn from comparisons between single samples of humans and single samples of apes, assuming that the performance of such limited samples is representative for the whole species. While we are definitely not the first to point out this issue (for detailed discussions, see, e.g., Leavens et al., 2019; Bard et al., 2021), we believe that it is important to reiterate and expose it, given its profound and pervasive consequences to the body of knowledge that we scientists create regarding human and ape nature. This single-group comparison may be less problematic when a human sample and an ape sample are found to perform similarly in a task, since this can safely lead to conclusions that the behaviour probed by the task is found, at least to some extent, in both studied species – although claims of universality would still be unwarranted. It is, however, much more problematic to draw conclusions concerning species-level *differences* from such limited data, given mounting evidence that the human data used to represent humanity in these studies is typically drawn from single WEIRD samples, thereby being, in fact, representative of only a minority (i.e., about 12%) of the world’s human population. Future research will benefit from conducting parallel within- and between-species comparisons based on data drawn from several populations, as recently illustrated by, e.g., Bard et al. (2021) in their proof-of-concept study of joint engagement. Alternatively, studies using the single-group approach should firmly limit their conclusions to the studied populations. Reaching clarity about the nature of social cognition in another species will require ensuring species-level representativeness before firm conclusions are drawn. In turn this will require considerable mapping work, where social behaviours are examined with adequately standardised methodology across a diversity of samples both from the wild and captivity. Such an approach will permit identifying both core – i.e., species-level – features, as well as variability, thereby providing indispensable insight to research on the ultimate and proximate mechanisms driving observed behaviours. Crucial in this respect is to take the causes for variability seriously, which calls for multidisciplinary treatment. Traditional experimental test psychology builds on success rates and population means. In some unlucky marriage between psychology and evolutionary theory it furthermore equates such scores with species traits. But theories about behaviour, and by extension cognitive evolution, cannot ignore what animals do, and which individuals do it. Variations in task engagement, causes for failure, previous experiences, etc., may be potentially relevant (see Persson, 2018 for an extended argumentation).

Considerable mapping work will also be required to design suitable and reliable methodologies that are, as much as possible, immune to human WEIRD biases. Currently, WEIRD biases affect research in human and comparative psychology by shaping both *what* is probed and *how* it is probed. Since for the past century, most psychological research – i.e., 96% based on the analysis of Arnett (2008) – has been based on the WEIRD minority, its thematic agenda has overwhelmingly prioritised constructs that are important or relevant in the socio-ecological niche of WEIRD populations. The conceptualisation and operationalisation of constructs deemed worthy of research efforts are also largely based on what is considered to be the norm in WEIRD subpopulations. This conceptual (and operational) norm, however, is far from being fully reflected in the behaviour of non-WEIRD samples. WEIRD operational criteria may not be satisfied by the performance of other populations, or the probed constructs may be altogether very weakly represented psychological phenomena (as reviewed above; for a more comprehensive review see also Henrich et al., 2010).

Overall, based on the currently available cross-cultural data, the WEIRD populations emerge as outliers on a range of measures concerning visual perception, spatial cognition and social cognition, including several of the traits addressed above – recursive mind reading, fairness, cooperation, conformity, and moral reasoning (Henrich et al., 2010; Bard et al., 2021). Defining human nature by behavioural outliers and measuring nonhuman social cognition with the WEIRD yardstick is, obviously, unlikely to lead to compelling conclusions about species-level differences. This exposes a comprehensive need to re-evaluate entrenched psychological constructs for their relevance to understanding human nature beyond WEIRD minorities, as well as to update the conceptualisation and operationalisation of relevant constructs so that they become useful research tools beyond WEIRD minorities. Unbiased research will require a thorough *decolonisation* of the field, as recently demonstrated by Bard et al. (2021). On the background of such concerns and desiderates, is the *shared intentionality* construct as outlined in SITh a defensible construct? As already reviewed in Section 3.1, Bard et al. (2021) attempted a decolonization of the construct of joint attention by conducting a detailed analysis of naturalistic observation data from three human socio-ecological settings. This analysis uncovered that SITh operational criteria for joint attention were not normative for the human species, being present in only 50% of identified bouts of triadic connectedness in the WEIRD sample and infrequent or even absent in other samples. The analysis also yielded an extended and more representative range of operational criteria for human joint attention. Using this extended range as a benchmark for the human species, Bard et al. (2021) found that ape versions of the competence at 12-months of age were not distinct from the human forms at 12-month of age. This study clearly illustrates both *how* to carry out the *decolonisation* of WEIRD-centric conceptualisations and operationalisations, as well as *why* this is needed for attaining more valid characterisations of socio-cognitive abilities at the species level – in both humans and other species – and thus more valid conclusions about species differences.

In our review, we have also highlighted several methodological biases that are associated with the WEIRD legacy. Experimental methods build on standardisation and strict control of measured variables to eliminate confounds and account for potential alternative interpretations. However, if standardisation and control are guided by operational criteria drawn from conceptualisations that are only relevant to a minority, the ensuing experimental design may be biased toward facilitating the performance of that minority. The task at hand may be optimally designed to engage targeted processes and motivations in a subpopulation, but not in others. For example, SITh conclusions about the lack of joint attention in apes rests primarily on data from object-choice tasks that require the subjects to either comprehend or deploy pointing. Yet this assessment tool represents a biased tool for probing the presence of joint attention not only in chimpanzees, but also in humans, since there are human groups that do not use pointing to direct others’ attention. If the goal is to address the presence of joint attention, a less biased approach would instead be one that implements functional (as opposed to formal) equivalence, whereby more ecologically valid indication behaviours replace the gesture ‘pointing’. Methodological concerns such as those outlined above are at the core of discussions regarding enculturation and socialisation effects and the ecological validity of research, i.e., whether findings obtained in the laboratory are replicable in naturalistic settings.

Through socialisation and enculturation, individuals acquire behaviours, customs, norms and values that are typical for their socio-ecological environment. In our review, for each thematic cluster, we have provided examples of behavioural variation that could be explained as socialisation and enculturation effects in both children and apes. In the comparative literature, a consistent finding is that apes raised in closer contact with (WEIRD) humans perform better in (WEIRD-based) socio-cognitive tests. While some would dismiss such enculturation effects as not representative for ape nature, the same argument can be made for WEIRD human children. They too exhibit WEIRD enculturation effects that are not necessarily representative of human nature. Moreover, we would like to argue that the enculturation effects found in some apes have scientific value for the understanding of human and ape nature. If WEIRD enculturation, without effort or training, unlocks in apes certain ways of behaving that have been characterised as uniquely human, then such ways of behaving can no longer be claimed to be uniquely human. Moreover, it highlights that WEIRD enculturation may provide WEIRD-enculturated individuals (whether children or apes) with substantial pre-test experience for certain task requirements (e.g., requirements to point, to share resources, or to reproduce behaviour), thereby enhancing their performance (for similar points and more examples, see, e.g., Leavens et al., 2019; Bard et al., 2021).

Concerns related to biases introduced by enculturation effects and ecological validity can also be raised with respect to shorter-term antecedents than enculturation. For example, it is customary in studies with human children to include an introductory phase aimed at familiarising participants with the experimenter(s) and/ or the testing environment. Such introductory phases may include playing games and are implicitly driven by the experimenters’ needs for social desirability, thereby cultivating a prosocial atmosphere and prosocial motivations prior to test start. This may constitute a source of motivational bias in both cross-cultural and comparative studies, in the sense that participants may be prompted to engage the task with completely different baseline motivations. In studies with apes, such introductory phases are largely absent, which may prime the apes to approach the task with purely instrumental rather than social motivations. This procedural difference may have significant consequences for test outcomes, considering evidence reviewed in Section 4.2, that prior experience with a prosocial agent may engender generalised prosociality in apes. In studies with non-WEIRD cultural samples, an introductory phase with a playful, overly friendly experimenter may inhibit (pro)social motivations and cultivate confusion, if deployed in socio-cultural settings where children do not play with or address unfamiliar adults (Berl and Hewlett, 2015).

Further highlighting concerns of ecological validity and, thus, concerns about the general validity of conclusions drawn from WEIRD-informed experiments, in our review we also discussed cases of systematic discrepancy between laboratory findings and findings from more naturalistic settings, both field studies and experiments. As we already pointed out, the artificial setup of experiments designed based on WEIRD assumptions risks generating sub-optimal situations for tests of social cognition. As detailed in 4.2.2, such artificial setups tend to disregard a range of social variables that mediate the expression of social behaviour not only in apes, but in humans as well. Overall, both short-term and long-term social (and cultural) antecedents ought to be considered when carrying out studies on socio-cognitive abilities. Once again, considerable mapping work is required to understand the dimensions of within-species socio-cognitive variability in both human and ape populations. As exemplified above with the study of Bard et al. (2021), this work will contribute to less biased experimental approaches, by providing input to experimental design and standardisation to ensure that experimental control and standardisation strike a suitable balance between formal and functional equivalence and does not end up throwing away the proverbial baby with the bathwater.

The claims of SITh have gained enormous popularity, especially beyond the field of comparative psychology. Yet, as we reviewed in this paper, several SITh arguments are only moderately supported by empirical data and the body of SITh-inconsistent findings continues to accumulate. SITh makes radical claims that a seeming divide separate selfish ape psychology from altruistic human psychology and that a whole suite of socio-cognitive traits and associated behaviours have uniquely emerged in the recent natural history of humans. Given the popularity of SITh, such claims may risk delegitimising comparative research on such traits. In addition, developmental psychological research, and research and intervention paradigms regarding clinical groups, such as autism spectrum disorders, are influenced by impactful psychological theories such as SITh. This alone emphasizes the importance to ‘get it right’. We hope that our review provides a compelling case that research with apes on the putatively human-unique features of social cognition that have come to be collectively designated as *shared intentionality* is more warranted than ever. We hope that our review also makes a compelling case that a thorough update is needed at the conceptual, operational, and methodological levels to align comparative cognition with the efforts deployed in cross-cultural psychology toward a WEIRD de-biased and de-colonised scientific endeavour.

## Author contributions

G-AS conceptualised and drafted the paper. TP critically reviewed the paper. All authors contributed to the article and approved the submitted version.
